# Accuracy of Electronic Health Record Data for Identifying Stroke Cases in Large-Scale Epidemiological Studies: A Systematic Review from the UK Biobank Stroke Outcomes Group

**DOI:** 10.1371/journal.pone.0140533

**Published:** 2015-10-23

**Authors:** Rebecca Woodfield, Ian Grant, UK Biobank Stroke Outcomes Group, UK Biobank Follow-Up and Outcomes Working Group, Cathie L. M. Sudlow

**Affiliations:** 1 Division of Clinical Neurosciences, Clinical Centre for Brain Sciences, University of Edinburgh, Edinburgh, United Kingdom; 2 Information Services Division, NHS, Edinburgh, United Kingdom; 3 UK Biobank, Adswood, Stockport, United Kingdom; University of Glasgow, UNITED KINGDOM

## Abstract

**Objective:**

Long-term follow-up of population-based prospective studies is often achieved through linkages to coded regional or national health care data. Our knowledge of the accuracy of such data is incomplete. To inform methods for identifying stroke cases in UK Biobank (a prospective study of 503,000 UK adults recruited in middle-age), we systematically evaluated the accuracy of these data for stroke and its main pathological types (ischaemic stroke, intracerebral haemorrhage, subarachnoid haemorrhage), determining the optimum codes for case identification.

**Methods:**

We sought studies published from 1990-November 2013, which compared coded data from death certificates, hospital admissions or primary care with a reference standard for stroke or its pathological types. We extracted information on a range of study characteristics and assessed study quality with the Quality Assessment of Diagnostic Studies tool (QUADAS-2). To assess accuracy, we extracted data on positive predictive values (PPV) and—where available—on sensitivity, specificity, and negative predictive values (NPV).

**Results:**

37 of 39 eligible studies assessed accuracy of International Classification of Diseases (ICD)-coded hospital or death certificate data. They varied widely in their settings, methods, reporting, quality, and in the choice and accuracy of codes. Although PPVs for stroke and its pathological types ranged from 6–97%, appropriately selected, stroke-specific codes (rather than broad cerebrovascular codes) consistently produced PPVs >70%, and in several studies >90%. The few studies with data on sensitivity, specificity and NPV showed higher sensitivity of hospital versus death certificate data for stroke, with specificity and NPV consistently >96%. Few studies assessed either primary care data or combinations of data sources.

**Conclusions:**

Particular stroke-specific codes can yield high PPVs (>90%) for stroke/stroke types. Inclusion of primary care data and combining data sources should improve accuracy in large epidemiological studies, but there is limited published information about these strategies.

## Introduction

Stroke is the second commonest cause of death worldwide and a major global cause of disability [1]. Pathological types and subtypes of stroke differ in their risk factor associations [2, 3]. Very large prospective studies, yielding large numbers of stroke cases, are needed to examine these associations reliably [4]. Linkage to routinely collected, coded healthcare data is a practical means of ascertaining stroke and other health-related outcomes. However, such data have variable completeness and accuracy [5–10].

UK Biobank is a very large prospective cohort study of 503,000 participants, aged 40–69 years when recruited in England, Scotland and Wales between 2006 and 2010 [11]. Participants completed a detailed questionnaire at baseline, underwent a range of physical measurements, and provided biological samples for genetic, biochemical and other analyses. Follow up is chiefly through cohort-wide linkages to National Health Service data, including electronic, coded death certificate, hospital, and primary care data. By 2017, around 5,000 incident strokes are expected to have occurred among UK Biobank participants [12].

**Fig 1:**
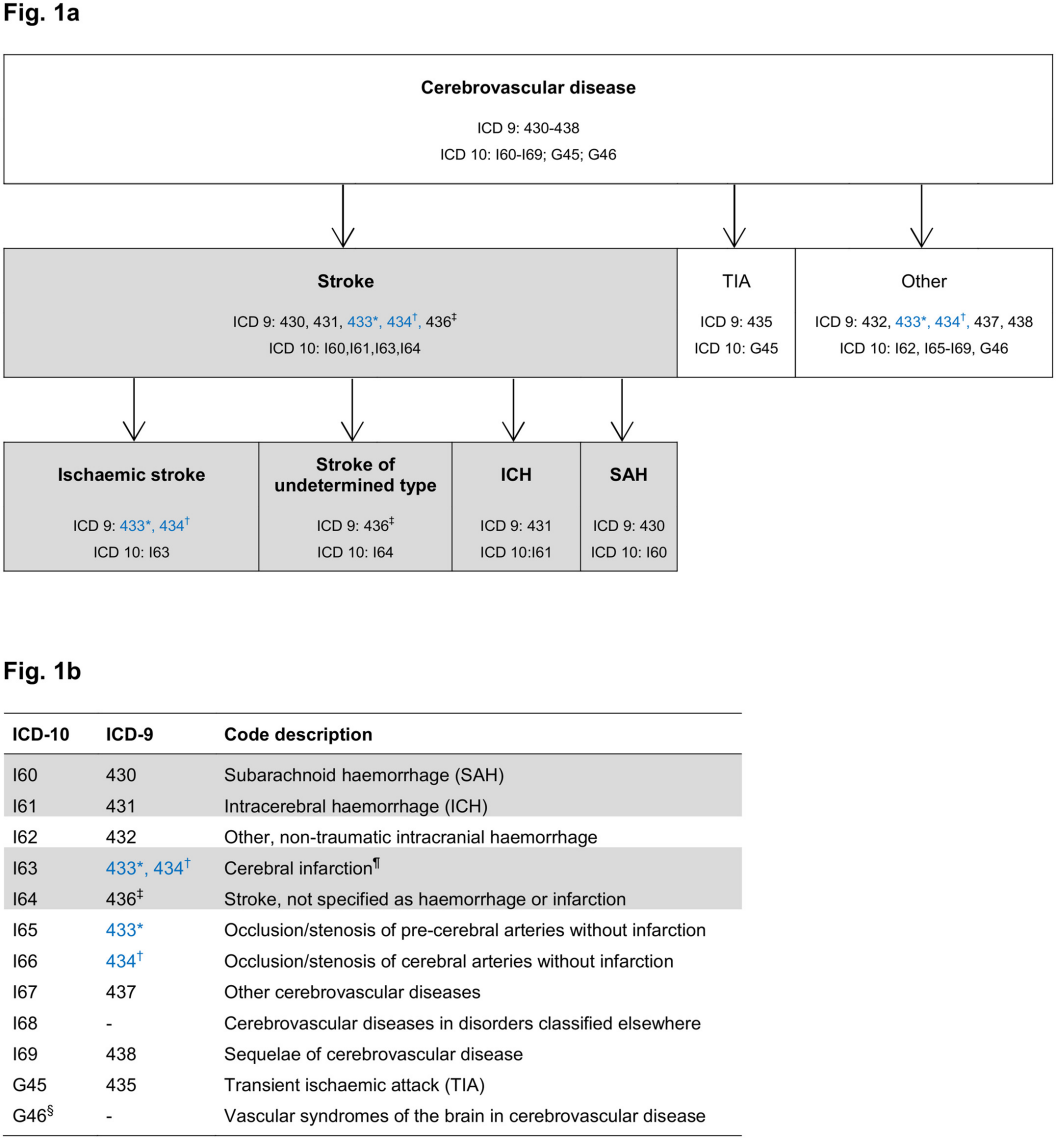
International Classification of Diseases (ICD) codes for cerebrovascular disease. * 433: occlusion/stenosis of pre-cerebral arteries *with or without infarction*. † 434: thrombosis/embolism of cerebral arteries *with or without infarction*. Codes in blue text denote ICD-9 codes which most closely represent stroke when subdivided using additional coding available in the clinically modified version of ICD-9 (ICD-9-CM) used in North America. In ICD-9-CM, *‘with infarction’* (433.x1, 434.x1) is distinguished from ‘*without infarction’* (433.x0, 434.x0). ‡ 436: acute, ill-defined cerebrovascular disease ¶ a pathological term for ischaemic stroke § G46: not a diagnostic code; may be used for the presenting symptoms of either stroke or TIA.

In most countries, including the UK, hospital admissions and death certificates are coded using the International Classification of Diseases (ICD) [13–15]. The primary ICD code identifies the main condition treated during a hospital admission, or the underlying cause of death. Secondary codes record additional diagnoses relevant to an admission, or contributing to death. Codes for cerebrovascular disease include a range of presentations. Fig 1 shows which ICD codes most closely match the World Health Organisation (WHO) definition of stroke [16] or of one of its three main pathological types: ischaemic stroke, intracerebral haemorrhage (ICH), and subarachnoid haemorrhage (SAH). Although not all of these represent a diagnosis of the clinical syndrome of stroke, many studies which have looked at determinants of stroke using linked ICD-coded datasets have included all cerebrovascular disease codes in the relevant ICD coding chapter, implicitly assuming that they are all codes for stroke. Over the last 10 years, health care systems in European countries have switched from ICD-9 to ICD-10, while those in North America use ICD-9-CM (a clinically-modified version of ICD-9). Primary care data in the UK are coded by general practitioners using the Read coding system, which encodes diagnoses, symptoms, signs, procedures, prescriptions and other administrative data [17, 18].

For health-related outcomes such as stroke, UK Biobank aims to maximise statistical power to detect genuine associations in nested case-control or case-cohort studies. This requires a strategy that identifies cases representative of the spectrum of the disease being studied with adequate sensitivity, and that maximises positive predictive value (PPV, the proportion of cases that are true positives). Minimising false positives will minimise loss of statistical power through misclassification of cases. Some false negatives can be tolerated, since these are diluted by the very much larger control population, with much more limited impact on statistical power. UK Biobank aims to fulfil these requirements by using multiple sources of coded data (primary care, hospital and death certificate data) to ascertain possible stroke cases, and then to implement algorithms, using combinations of coded data, supplemented where required by more detailed medical record review, to confirm and sub-classify cases of stroke. An important first step in developing such algorithms is to understand the accuracy of the coded data sources. To inform approaches to ascertainment, confirmation and sub-classification of stroke in UK Biobank and other large epidemiological studies, we therefore performed a systematic review of published studies of the accuracy of coded health record data for stroke and its main pathological types. We chose not to include transient ischaemic attacks (TIAs), which are clinically harder to diagnose accurately, with poor agreement even amongst experts [19], and of substantially less public health impact than strokes. We used the traditional, epidemiological ‘symptom-based’ definition of stroke (symptom duration >24 hours) to distinguish stroke from TIA.[16] The more recent, alternative ‘tissue-based’ definition relies on the presence of brain infarction to diagnose stroke, irrespective of symptom duration (<24hours).[20] Accurate diagnosis of brain infarction depends on the availability, choice, and timing of brain imaging, which may vary between different centres.[21] We chose to use the ‘symptom-based’ definition to maximise comparability between different studies.

## Methods

The study protocol is displayed in S1 Appendix.

### Search Strategy

We searched Medline and Embase from 1990 to November 2013 for studies which compared electronic health record data coded events against a reference standard data source for stroke or its main types. We used a combination of medical subject heading and text word terms for ‘cerebrovascular disease’, ‘stroke’, ‘medical records’, ‘clinical coding’, and ‘validation studies’ (S1 Appendix). We identified additional relevant studies by reviewing the bibliographies of included primary studies and relevant reviews, as well as lists of publications from the Clinical Practice Research Datalink [22] and The Health Improvement Network [23] websites for studies evaluating accuracy of primary care data.

### Eligibility Criteria

Included studies had to have assessed International Classification of Diseases (ICD) or Read coded events against a reference standard data source for stroke or of one or more of its three major pathological types (Fig 1), defined according to WHO or equivalent definitions.[16] Studies had to report which codes were validated and either their positive predictive value (PPV) or data from which it could be calculated. We excluded studies with less than 50 coded events (since these would have limited precision) and studies in highly selected populations (e.g., those with vascular risk factors or known vascular disease) at increased risk of stroke because of the influence of stroke prevalence on PPV. One author reviewed all titles and abstracts to select potentially relevant studies, and a subset of 10% of titles and abstracts was independently reviewed by a second author, who reached the same conclusions as the first. Two authors independently reviewed full texts of potentially relevant studies and selected studies for inclusion. Any areas of uncertainty from this two phase study selection process were discussed and resolved with a third, senior author with extensive experience both in stroke epidemiology and in systematic review methodology.

### Data Extraction and Analysis

We extracted and tabulated information from each included study on: first author and publication year; geographic setting (country); age (mean and/or range) of included cases (coded events); data source (hospital, death certificates, primary care); coding system and version; codes used to identify cases; diagnostic position of these codes in the electronic health record (primary versus secondary); number of cases (coded events) compared against the reference standard; reference standard used; PPV and, where reported or calculable, sensitivity, specificity, and negative predictive value (NPV) of codes. We only extracted sensitivity, specificity and NPV values where the reference standard was a population-based stroke register which had clearly aimed to include all stroke cases in the population under study.

We assessed study-level quality with a modified version of the Quality Assessment of Diagnostic Studies tool (QUADAS-2),[24] adapted from a recent systematic review of the validity of myocardial infarction diagnoses in administrative databases.[25] We used this to assess reporting quality, generalisability to the UK population (because we sought to recommend codes for UK Biobank), and risk of bias. The study protocol (S1 Appendix) provides a detailed list of questions and scoring methods. An overall quality score (0–14) was derived by combining scores for reporting quality, generalisability, and low risk of bias. We did not exclude studies on the basis of quality assessments.

We calculated 95% confidence intervals for PPV, sensitivity, specificity and NPV values in *Stata* (version 12) using the Wilson method for binomial proportions [26]. For stroke and each of its main pathological types, we assessed the influence on PPV (and, where available, sensitivity) of the codes used to identify stroke cases, and of other study characteristics, using visual inspection of tabulated data and forest plots, and making within-study comparisons where possible to minimise bias. We did not undertake formal meta-analyses or meta-regression because of the substantial heterogeneity between studies in their settings, methods and reporting.

## Results

### Studies Identified

**Fig 2:**
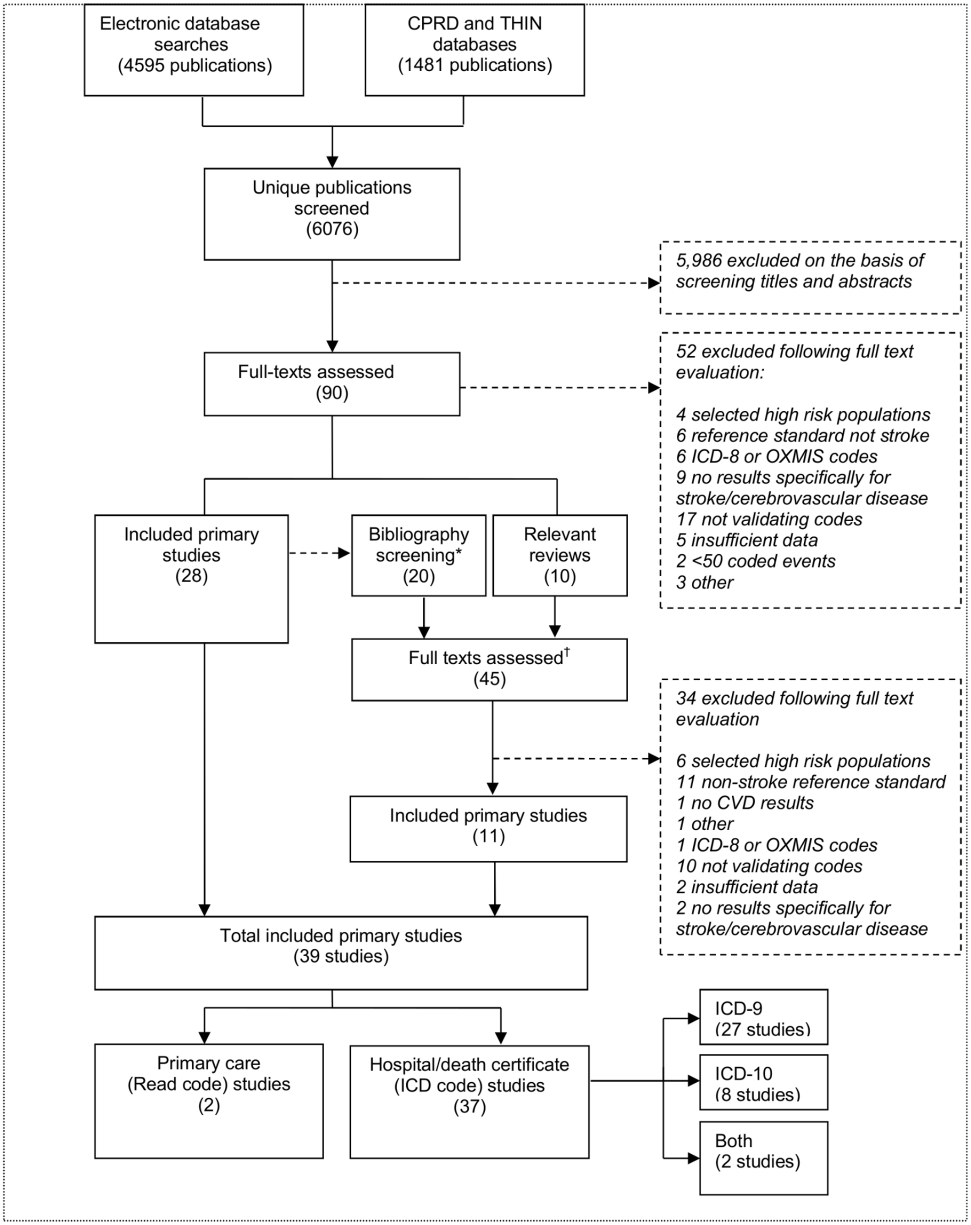
Selection of studies. *Additional studies identified from bibliography screening. †Additional studies identified from review articles and bibliography screening.

A total of 39 studies fulfilled our inclusion criteria (Fig 2). Of these, 37 were of ICD-coded hospital data, death certificates, or both. Only two were of Read-coded primary care data [27, 28].

### Characteristics of Studies of Hospital and Death Certificate (ICD-Coded) Data

Study characteristics are displayed in S1 Table. The 37 studies were from North America [29–43], Europe [44–64] (eight UK based [56–63]) and Australia [65]. They assessed ICD code versions 9 [29–43, 48–52, 55–62], 10 [44–46, 53, 54, 56, 57, 63], or both [47, 65]. Most studies used hospital data only, but one was of death certificates only [65], and six used both [40, 45, 47, 50, 55, 56]. The majority of studies sought cases of stroke [29, 31–39, 44–46, 48, 49, 51, 54–56, 58–62, 65], 14 sought ischaemic stroke [33, 34, 40–44, 46–49, 52, 53, 57], five haemorrhagic stroke (ICH or SAH) [33, 44, 47–49], four ICH [47–49, 63], and four SAH [40, 47, 57, 63].

The range of codes used to ascertain events within each of these categories varied widely. To identify stroke cases, the largest number of studies used the whole range of cerebrovascular disease codes (either with or without codes for transient ischaemic attack [TIA]) [31–33, 37–39, 44, 46, 48–51, 55, 56, 61, 62, 65], but others used stroke-specific codes [34, 44, 46, 48, 49]. Several others used various miscellaneous groups of cerebrovascular codes to identify stroke cases [29, 30, 35, 36, 58, 59], while a further four did not include SAH in their definition of stroke and so excluded SAH codes [45, 54, 60, 64]. The diagnostic position of codes was recorded by 31 studies, of which 11 used the primary position alone [34, 36, 38, 39, 42, 53, 54, 58, 61–63]. Reference standards were either review (generally by a specialist physician) of the hospital or primary care records or a hospital discharge summary [29–44, 46, 51, 53, 56, 57, 62, 63, 65], or comparison with a population- or hospital-based stroke register [31, 32, 45, 47–50, 52–55, 58–61, 64]. Stroke register cases were identified using administrative data (generally multiple overlapping sources) [45, 47, 48, 50, 52, 54, 55, 58, 64], with ‘hot pursuit’ [32, 45, 48, 52–54, 58, 64], and confirmation by expert medical record review.

### Quality Assessment

Detailed results of the quality assessment are displayed in S2 Table. Quality scores ranged from 4 to 12 (median 9, interquartile range 8 to 11). With respect to reporting quality, participant selection criteria and coding algorithms were generally well reported, but only ten studies acknowledged the potential for uncertainty of the reference standard diagnosis in their results. [33, 36, 38, 39, 41, 45, 56, 58, 59, 64] With respect to generalisability to the UK population, only eight studies were conducted in the UK. However, all the other studies were based in high income countries, among populations of predominantly European origin with broadly similar health care provision, and are therefore likely to be broadly generalizable (from a global perspective) to population-based studies in these types of settings (including the UK). Of the UK-based studies, two had suboptimal generalisability because all coded discharges were taken from a single hospital department, [61, 62] while for the other six generalisability was unclear due to incomplete reporting.[56–60, 63]

With respect to risk of bias, only five studies achieved the optimum score.[33, 45, 50, 54, 65] Incomplete reference standard data (due to a variable proportion of missing or irretrievable records) [29–31, 34, 36, 37, 39, 42–44, 46–48, 51, 52, 55–57, 60, 63, 64] and lack of or inadequate blinding of adjudicators to the coded diagnosis [29, 30, 32, 34, 36–39, 42–45, 47–49, 51–53, 56, 57, 61, 62] were the most common potential causes of bias.

### Accuracy of ICD-Coded Events

The range of PPVs reported for various codes used to identify stroke or one of its main pathological types was very broad, reflecting considerable heterogeneity of study characteristics. Results were particularly variable for all stroke (PPV 31–97%) and for ischaemic stroke (PPV 6–95%), while they appeared more consistent for haemorrhagic stroke (PPV 73–89%), SAH (PPV 86–96%) and ICH (PPV 71–96%), although based on fewer studies.

#### Within-study comparisons

Only six studies used a population-based reference standard and, of these, only four (all from Scandinavian countries) [45, 48, 50, 64] provided sufficient data to calculate sensitivity, specificity and negative predictive value (NPV) of codes for stroke. Sensitivities for identifying stroke were around 80% or more using general cerebrovascular or stroke-specific codes from either hospital data or hospital data combined with death certificates, but—unsurprisingly—sensitivity was much lower for death certificates alone (S3 Table). There were no data on sensitivity for the main pathological types of stroke.

Where calculable, specificity and NPV were uniformly high (range 96–99.9%), reflecting the relatively small proportion of false negative strokes (amongst all non-stroke and code negative numbers, respectively). [45, 48, 50, 64]

Several within-study comparisons showed that the groups of codes with the highest PPVs (68–90%) for all types of stroke combined were 430, 431, 434, 436 (ICD-9) or I60, I61, I63, I64 (ICD-10) (Table 1). Compared with general cerebrovascular codes (ICD-9 430–438, or ICD-10 I60-I69+/-G45), selection of these stroke-specific codes gave consistently higher PPVs (absolute increase of 17–30%) (Table 1). Stroke-specific codes inevitably identified fewer coded events than general cerebrovascular ones (numbers fell by a third to over a half, Table 1), but the impact on sensitivity appeared limited (absolute decrease of 5%) in the one study that provided data on this [48].

**Table 1:** Effect on PPV of codes used to identify stroke: within-study comparisons*.

Study	Codes	Code definition / diagnosis sought	Coded events	PPV (%, & 95% CI)
Johnsen [44] 2002	I60-I69 + G45	CVD	565	58 (58–62)
	I60, I61, I63, I64	Stroke	378	79 (75–83)
Krarup [46] 2007^†^	I60-I69 + G45	CVD	236	69 (59–71)
	I60, I61, I63, I64	Stroke	164	86 (76–88)
Ellekjaer [48] 1999	430–438	CVD	759	49 (45–52)
	430, 431, 434, 436	Stroke	508	68 (64–72)
Leone [49] 2004	430–438	CVD	1017	60 (57–63)
	430, 431, 434, 436	Stroke	411	90 (87–93)

*If there was more than one result per code, results are shown for the largest number of cases assessed.

^**†**^Mean PPV taken from range of values in original publication.

For identifying ischaemic stroke, codes I63 (ICD-10) or 434 (ICD-9) achieved reasonably high PPVs (range 66 to 88%) [33, 42–44, 49, 53, 57], while code 433 (ICD-9) performed consistently poorly in studies which assessed it (PPV range 6% to 14%) [33, 43, 49]. The addition of codes for unspecified type of stroke (436 [ICD-9] or I64 [ICD-10]) to ischaemic stroke codes increased the number of coded events identified within each study, with in general either no change or only a few % absolute decrease in PPV (Table 2) [33, 42–44, 46–49, 52, 57].

**Table 2:** Effect on PPV of codes used to identify ischaemic stroke: within-study comparisons*.

Study	Codes	Code definition / diagnosis sought	Coded events	PPV (%, & 95% CI)
Johnsen [44]	I63	Ischaemic stroke	113	88 (80–93)
2002	I64	Unspecified stroke	200	70 (63–76)
	I63, I64	Ischaemic and unspecified stroke	313	76 (71–80)
Wright [57]	I63	Ischaemic stroke	190	86 (81–91)
2012	I64	Unspecified stroke	119	66 (57–73)
	I63, I64	Ischaemic and unspecified stroke	309	78 (73–83)
Ellekjaer [48]	434	Ischaemic stroke	313	66 (60–71)
1999	436	Unspecified stroke	89	62 (51–71)
	434, 436	Ischaemic and unspecified stroke	402	65 (60–69)
Leone [45]	434	Ischaemic stroke	202	87 (82–91)
2004	433	Ischaemic stroke	134	6 (3–11)
	436	Unspecified stroke	57	70 (57–80)
	434, 436	Ischaemic and unspecified stroke	259	83 (78–87)
	433, 434, 436	Ischaemic and unspecified stroke	393	57 (52–62)
Rosamond [33]	434	Ischaemic stroke	186	77 (70–82)
1999	433	Ischaemic stroke	266	14 (10–18)
	436	Unspecified stroke	108	70 (52–76)
	434, 436	Ischaemic and unspecified stroke	294	73 (68–78)
	433, 434, 436	Ischaemic and unspecified stroke	560	45 (41–49)
Benesch [43]	434	Ischaemic stroke	226	85 (79–89)
1997	433	Ischaemic stroke	295	6 (4–9)
	434, 436	Ischaemic and unspecified stroke	250	86 (82–90)
	433, 434, 436	Ischaemic and unspecified stroke	550	43 (38–47)
Krarup [46]	I64	Unspecified stroke	105	60 (50–69)
2007	I63, I64	Ischaemic and unspecified stroke	138	70 (61–77)
Rinaldi [52]	436	Unspecified stroke	177	71 (64–77)
2003	434, 436	Ischaemic and unspecified stroke	180	71 (64–77)
Tonolen [48]	433, 434, I63	Ischaemic stroke	2711	82 (81–83)
2007	433, 434, 436, I63, I64	Ischaemic and unspecified stroke	2900	83 (82–84)
Goldstein [42]	434	Ischaemic stroke	108	82 (74–88)
1998	434.x1	Ischaemic stroke	106	82 (74–88)
	434, 436	Ischaemic and unspecified stroke	127	82 (74–88)
	433, 434, 436	Ischaemic and unspecified stroke	175	61 (53–68)

*If there was more than one result per code, results are shown for the largest number of cases assessed.

^**†**^Mean PPV taken from range of values in original publication.

Eight studies (all of ICD-9 codes) assessed influence of coding position on PPV for a variety of ICD-9 code groups (cerebrovascular disease codes, ischaemic stroke codes, or haemorrhagic stroke codes) [30, 31, 34, 37, 40, 43, 49, 52]. Restriction to the primary position code (versus inclusion of codes from the primary or secondary diagnostic position) increased the PPV, but by no more than about 5–10% in all but two studies [30, 37] (S4 Table). It was not possible directly to assess the influence of code position on sensitivity, but restriction to the primary position reduced the number of coded events identified by around 10–30%.

#### Comparisons between groups of studies reporting PPV for stroke and its main types

**Fig 3:**
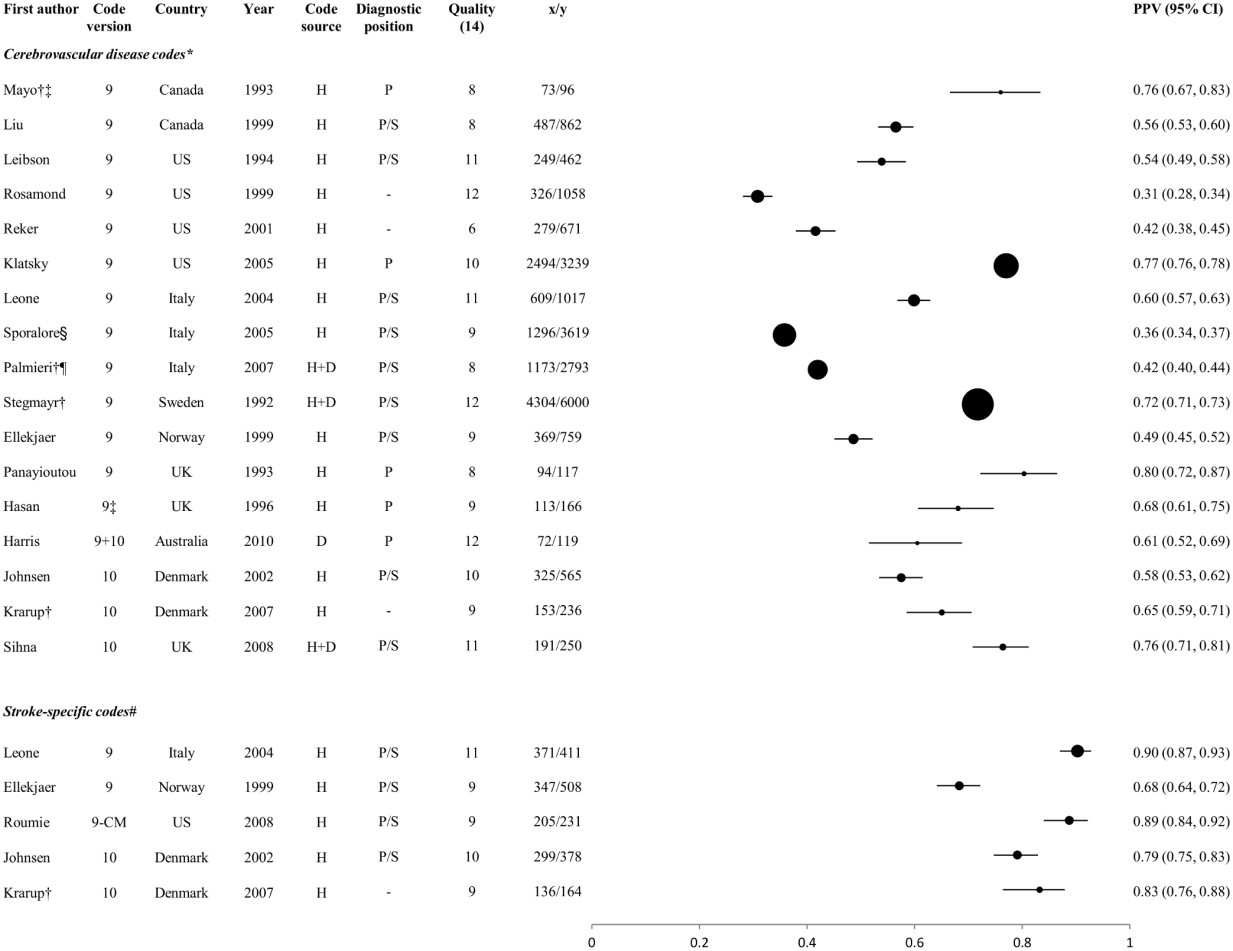
Positive predictive values of codes for stroke. H: hospital data, D: death certificates, H+D: hospital data and death certificates; x = number of coded events confirmed as ‘true cases’ by the reference standard; y = total number of coded events; x/y = PPV. Circles represent PPVs, and horizontal lines denote 95% confidence intervals (CIs). Circle size is proportional to the inverse variance of the PPV. Where more than one result was available for a particular study, the result for the largest number of coded events validated is shown. * Cerebrovascular codes: I60-I69+/-G45 (ICD-10) or 430–438 (ICD-9), unless otherwise specified † Mean PPV (taken from the range published in the study) ‡ Excluding codes 435 (TIA) and 438 (sequelae of cebrovascular disease) § Excluding code 435 (TIA) and including code 342 (hemiplegia and hemiparesis) ¶ Excluding code 435 (TIA) # Stroke-specific codes: 160, 161, 163, 164 (ICD-10), 430, 431, 434, 436 (ICD-9), 430, 431, 433.x1, 434.x1 (ICD-9-CM). ¥ Ischaemic stoke and unspecified stroke codes: I63, I64 (ICD-10), 434, 436 (ICD-9), 433.x1, 434.x1, 436 (ICD-9-CM) **Ischaemic stroke codes:163 (ICD-10), 434 (ICD-9), 433.x1, 434.x1 (ICD-9-CM) †† Haemorrhagic stroke codes:I60, I61 (ICD-10), 430, 431 (ICD-9) ‡‡ Subarachnoid haemorrhage stroke codes:I60 (ICD-10), 430 (ICD-9) ¶¶ Intracerebral haemorrhage stroke codes:I61 (ICD-10), 431 (ICD-9)

**Fig 4:**
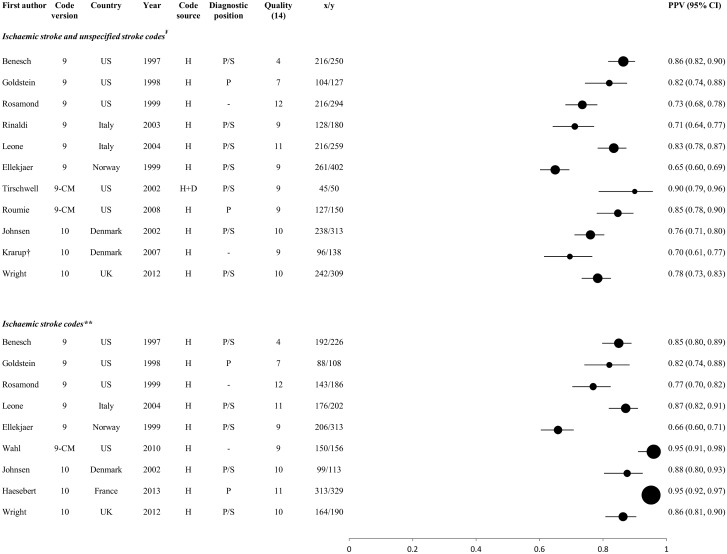
Positive predictive values of codes for ischaemic stroke. H: hospital data, D: death certificates, H+D: hospital data and death certificates; x = number of coded events confirmed as ‘true cases’ by the reference standard; y = total number of coded events; x/y = PPV. Circles represent PPVs, and horizontal lines denote 95% confidence intervals (CIs). Circle size is proportional to the inverse variance of the PPV. Where more than one result was available for a particular study, the result for the largest number of coded events validated is shown. † Mean PPV (taken from the range published in the study) ¥ Ischaemic stoke and unspecified stroke codes: I63, I64 (ICD-10), 434, 436 (ICD-9), 433.x1, 434.x1, 436 (ICD-9-CM) **Ischaemic stroke codes:163 (ICD-10), 434 (ICD-9), 433.x1, 434.x1 (ICD-9-CM)

**Fig 5:**
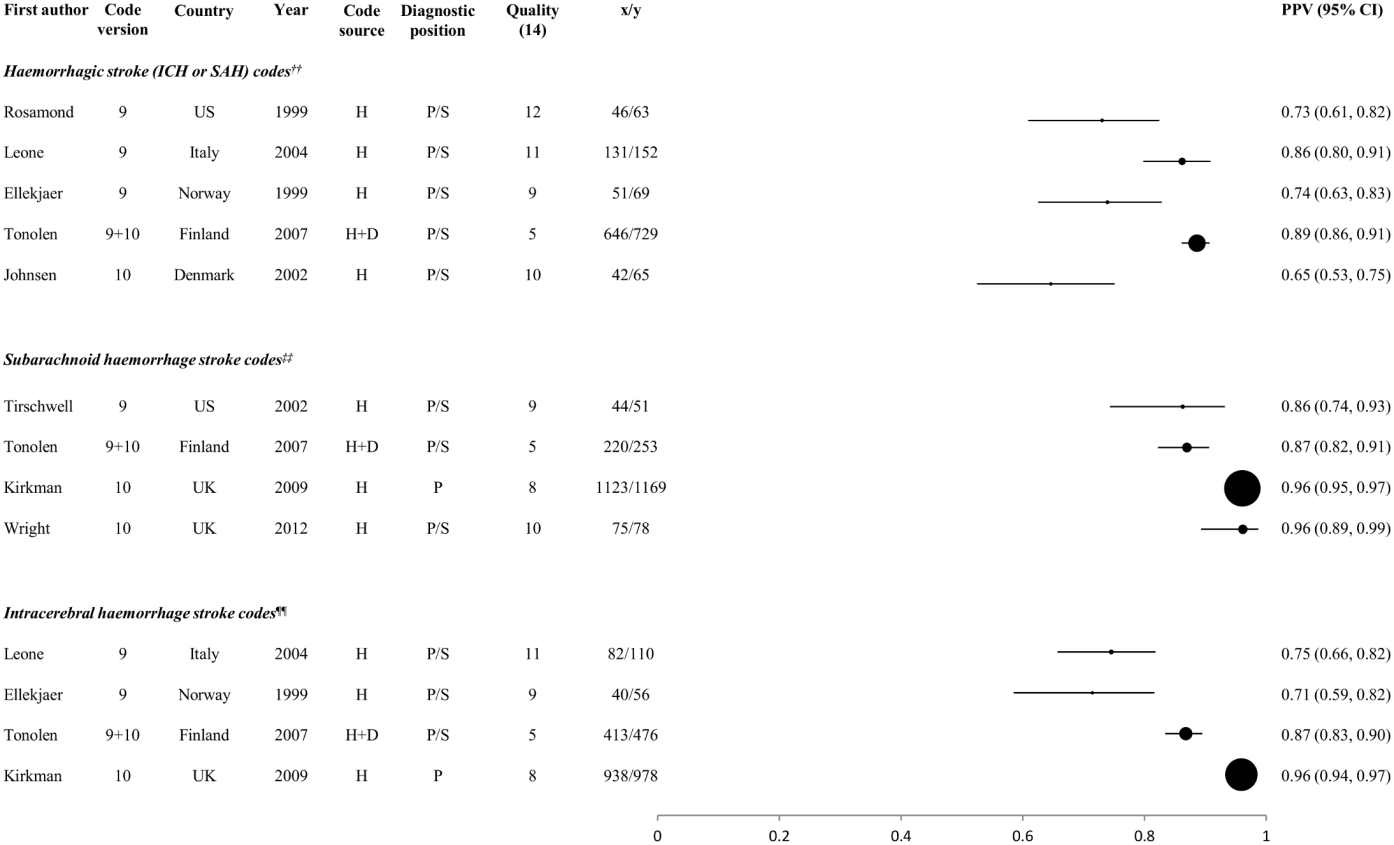
Positive predictive values of codes for haemorrhagic stroke. H: hospital data, D: death certificates, H+D: hospital data and death certificates; x = number of coded events confirmed as ‘true cases’ by the reference standard; y = total number of coded events; x/y = PPV. Circles represent PPVs, and horizontal lines denote 95% confidence intervals (CIs). Circle size is proportional to the inverse variance of the PPV. Where more than one result was available for a particular study, the result for the largest number of coded events validated is shown. † Mean PPV (taken from the range published in the study) †† Haemorrhagic stroke codes:I60, I61 (ICD-10), 430, 431 (ICD-9) ‡‡ Subarachnoid haemorrhage stroke codes:I60 (ICD-10), 430 (ICD-9) ¶¶ Intracerebral haemorrhage stroke codes:I61 (ICD-10), 431 (ICD-9)

The PPV of codes for stroke and its main types, stratified according to the code group(s) selected (see below), are displayed in Figs 3–5. They display results of studies which identified: stroke events using either a broad selection of cerebrovascular codes or stroke-specific codes (Fig 3); ischaemic stroke events, using either codes for ischaemic and unspecified type of stroke or for ischaemic stroke alone (Fig 4.); and haemorrhagic stroke events using codes for ICH and SAH together or separately (Fig 5.). Informed by our within-study comparisons, results exclude studies which included the poorly performing ICD-9 code 433 among the stroke-specific or ischaemic stroke codes, except those which used the clinical modification 433.x1 (Fig 1, Table 2, Fig 4).

For each of stroke and its main pathological types, PPVs of >90% were achieved in some studies (Figs 3–5). In line with results from within-study comparisons (Table 1), stroke-specific codes yielded higher PPVs for stroke (range 68–90%) than general cerebrovascular disease codes (range 31–80%) (Fig 3), while PPVs for ischaemic stroke were slightly higher with codes for ischaemic stroke alone (range 66–95%) than with codes for ischaemic and unspecified stroke (range 65–90%), but identified smaller numbers of outcomes (Fig 4). Codes for haemorrhagic stroke, and for ICH and SAH separately, performed consistently well or very well (PPV range 65–96%) (Fig 5). In general, ICD-10 appeared to perform better than ICD-9 codes, except where the ‘clinical modification’ (ICD-9-CM, see Fig 1) was available. Studies from the UK, yielding data that might be considered most informative for UK Biobank, reported PPVs of 78% and 86% for ischaemic stroke in one study [57] (the lower value when codes for unspecified stroke were included), 96% for SAH in two studies [57, 63] and 96% for ICH in one study [63]. The quality scores did not appear to influence PPV (Figs 3–5).

#### Selection of the best code using a code hierarchy

Two studies used a ‘code hierarchy’ to select a single stroke code when more than one was used for an individual hospital admission [34,40]. These studies selected the single ‘best code’ for each case, based on presumed coding accuracy (SAH>ICH>ischaemic stroke>transient ischaemic attack [TIA]). This approach was no more accurate than selection of the primary position code in one study [40], and less accurate than selection of the primary position code in another [34].(S4 Table).

#### Distinguishing ischaemic stroke subtypes

Very few studies assessed accuracy of ICD codes for more detailed ischaemic stroke subtypes, and none assessed accuracy for subtypes of SAH or ICH.

One study found that out of 106 coded events for ischaemic stroke subtypes, >70% had unspecified ischaemic stroke subtype codes [42]. The PPV of the cardiac embolism subtype code was 73% (based on only 11 coded events), but PPVs for other ischaemic subtypes were not reported.

Another study attempted to classify ischaemic strokes into four subtypes (lacunar stroke, cardiac embolism, large artery atherosclerosis and other) based on the hospital discharge abstract (which was used to generate the ICD codes) rather than the codes themselves [54]. This approach produced PPVs of 66–87% (highest for cardiac embolism and lacunar ischaemic stroke), and sensitivities of 67–74% (highest for cardiac embolism and large artery atherosclerosis).

### Studies of Read-Coded Primary Care Data

Two UK-based studies reported PPVs of Read codes from primary care data, one for ischaemic and one for haemorrhagic stroke (S5 Table) [27, 28]. Neither study reported code sensitivity. PPV was 89% for ischaemic stroke and 82% for haemorrhagic stroke, increasing to 90% for haemorrhagic stroke with exclusion of haemorrhagic codes which overlapped with antithrombotic drug prescription codes.

### Combining Multiple Data Sources

None of the included studies assessed the combination of primary care codes with hospital or death certificate codes for stroke or its main types. A few excluded studies compared primary care and hospital codes to search for stroke plus TIA [66, 67]. A UK study found that, compared to hospital ICD codes for stroke plus TIA in a primary care population of ~5800 individuals, Read codes increased sensitivity and decreased PPV by absolute values of 53% and 17% respectively [66]. Similarly, a community-based study in Canada found that combining primary care physician billing data with hospital ICD codes detected more stroke/TIA events, but with lower PPV, compared to ICD codes alone: sensitivity for combined data sources was 78% (95% CI 66%-83%) versus 37% (95% CI 28%-46%) for ICD codes alone; PPV for combined data sources was 40% (95% CI 33%-46%) versus 81% (95% CI 70%-92%) for ICD codes alone [67].

Two UK studies explored the possibility of using medical record extracts to reduce the proportion of unspecified stroke codes (I64) [57, 68]. In one, the primary care record held information to classify 74% of ICD-coded ‘unspecified strokes’ as ischaemic or haemorrhagic [57]. In the other, CT brain scan reports were used to assign ~ 8400 stroke cases (identified by ischaemic stroke, intracerebral haemorrhage or unspecified stroke codes) to a main pathological type [68]. The proportion of ‘unspecified’ stroke cases fell from 67% to 33% when ICD coded data plus natural language processing of scan reports was used, versus ICD coded data alone. Using a physician’s classification of radiology reports of 300 randomly selected cases as a reference standard, ICD coding plus analysis of scan reports was more accurate for ischaemic (PPV 95%, 95% CI 90% to 97%) than for haemorrhagic stroke (PPV 77%, 95% CI 69% to 73%).

## Discussion

As far as we are aware from published work, this is the first systematic assessment of the accuracy of coded hospital, death certificate and primary care data for identifying stroke. Previous reviews have been less comprehensive in their data presentation and analysis, or less precise in their definition of stroke, with the inclusion of TIA, subdural haemorrhage, or all cerebrovascular disease in the reference standard. A previous review based on US studies alone reported similar results but did not include UK-based studies or consider either ICD-10 codes or the performance of primary care data or combined data sources [10]. Previous UK-based reviews of ICD or Read code accuracy have reviewed overall accuracy for a wide range of diseases rather than accuracy for stroke specifically [69, 70], with limited numbers of stroke/cerebrovascular disease studies [9, 71–73].

We found wide variation in the performance of ICD codes for stroke and its main types, reflecting the heterogeneity of codes assessed and variation in study settings and methods. Our data also show a lack of consensus among stroke epidemiology studies about which codes should be used for identifying stroke outcomes. We have demonstrated that with appropriate selection of stroke-specific codes, PPVs of close to or >90% can be achieved for stroke and each of its main pathological types. Such PPVs will be adequate for many large scale epidemiological studies of the determinants of stroke. However, we found very few studies of the accuracy for stroke of Read-coded primary care data or of two or more overlapping data sources. Furthermore, the few available studies of ICD-coded data sources for identification of ischaemic stroke subtypes found that the majority of ischaemic subtype codes were ‘unspecified’[42], and reliability of ischaemic subtype classification was limited [74, 75]. We found no studies of the accuracy of coded data for identification of subtypes of ICH or SAH.

Within- and between-study comparisons revealed several consistent patterns. First, for stroke of any pathological type, PPV is increased by use of stroke-specific rather than general cerebrovascular codes, making it preferable to use stroke-specific codes to maximise PPV if no further adjudication of outcomes is planned after identification using ICD codes. Limited evidence suggests that sensitivity is poor when only death certificate data are used as a data source and is markedly increased by including data from hospital admissions, without compromising PPV.[45, 64] Based on one study, using general cerebrovascular rather than stroke-specific codes also seems likely to increase sensitivity, albeit perhaps by only a small amount and at the expense of a lower PPV.[48] To reduce the number of false positives, this method of identifying stroke outcomes is, therefore, probably best used in combination with further steps to confirm which cases are true positives. The best approach for this confirmation process requires further investigation, but could potentially use combinations of ICD codes with coded data from primary care or other sources, or more detailed medical record review. Second, for ischaemic stroke, a greater number of outcomes are identified with little reduction in PPV by using a combination of ischaemic and unspecified stroke codes to identify outcomes. Third, specific codes for ICH and SAH were found to have generally high PPVs (range 71 to 96%). Fourth, across a range of codes for cerebrovascular disease, stroke and pathological stroke types, identification of stroke outcomes using only codes in the primary position increased PPV, but generally by only a modest amount and at the expense of missing true positive outcomes. Furthermore, the relevant studies were of ICD-9 codes only, which are now rarely used outside the USA.[31, 32, 34, 37, 40, 43, 49, 52] Thus, use of appropriately selected codes in both the primary and secondary positions would seem appropriate for most purposes.

There were some limitations. First, since we only searched two online databases, we may have missed a few relevant articles. However, we also reviewed bibliographies of all included publications to increase the sensitivity of our search strategy. Second, our finding that use of the primary diagnostic position improved PPV in some studies may have been due to publication or reporting bias, since many studies did not report on this. Third, since PPV increases with increasing prevalence of the outcome studied, the lower prevalence of ICH and SAH (which together comprise around 20% of all strokes) compared with ischaemic stroke means that the PPVs of these different pathological types are not directly comparable. Fourth, some included studies had potentially less accurate sources available as a reference standard, such as hospital discharge summaries (a free text summary of the hospital admission, which is often written by less experienced doctors), or non-specialist primary care records (potentially based on hospital discharge summaries). We may have overestimated PPV of codes for haemorrhagic stroke types by using such reference standard data from two UK-based studies [57, 63]. Apart from the examples above, all included studies used more accurate reference standard data sources (independent medical record review and/or expert-led stroke registers), and we excluded studies which did not use WHO or equivalent definitions of stroke and its main types.[16] However, there is no ‘gold standard’ diagnosis for stroke. Even experts are inconsistent in their ability to diagnose stroke,[76], and choice and timing of imaging (which may vary between centres and therefore between studies) influences the diagnostic accuracy of stroke types.[77, 78] Fifth, the paucity of specific published data about the accuracy of Read-coded primary care data for stroke is an important further limitation, since up to half of stroke patients are not admitted to hospital in the UK [79, 80], and hospitalised and non-hospitalised strokes may differ in the distribution of pathological types and subtypes and in their risk factor associations [81]. Combining primary care data with other sources (hospital and death certificate data) should improve the detection of non-hospitalised cases, reducing potential bias in the selection of cases. Although we identified six systematic reviews of Read code accuracy for a wide range of diseases [9, 69–73], none included data specifically for stroke. Two excluded studies validated Read codes for cerebrovascular disease [66, 82], against a reference standard diagnosis of ‘cerebrovascular disease’. These ‘reference standards’ were potentially less accurate because they included hospital ICD codes and patient-self-report without medical record review, or used internal validation by GP questionnaire (not an independent data source). In addition to improving case ascertainment, primary care data may enhance the sub-classification of potential stroke cases. Around 40% of ICD codes for stroke are of unspecified type, although this proportion may be declining [83, 84]. Diagnostic codes combined with investigation, procedure, and/or medication codes (in primary care or hospital data) may increase PPV for ischaemic or haemorrhagic stroke [28, 53].

## Conclusions

Informed by this review, we recommend using 430, 431, 434, 436 (ICD-9), or I60, I61, I63, I64 (ICD-10), in either the primary or secondary diagnostic position to identify stroke cases with sufficiently high PPV for use in epidemiological studies where further confirmation steps are not envisaged. This may achieve PPVs of >90% for stroke. To increase the number of potential events identified, we suggest using all cerebrovascular disease ICD codes (ICD-9 430–438, or ICD-10 I60-I69, G45, G46) in both primary and secondary positions, but these would have to be combined with additional methods of stroke confirmation to maintain a high PPV. For ischaemic stroke we recommend codes 434, 436 (ICD-9), 433.x1, 434.x1, 436 (ICD-9-CM), and I63, I64 (ICD-10). For haemorrhagic stroke we recommend 430 (ICD-9) and I60 (ICD-10) for SAH, and 431 (ICD-9) and I61 (ICD-10) for ICH. Identifying more detailed stroke subtypes is likely to require coded data from investigations, procedures, and/or drug prescriptions, as well as diagnostic codes, and possibly more detailed review of medical record and imaging data.

Ultimately, UK Biobank aims to improve the accuracy and completeness of stroke outcomes ascertainment by linking multiple sources of coded data. Further work is needed to examine the use of multiple coded data sources to maximise PPV and sensitivity for stroke.

## Supporting Information

S1 Appendix Study protocol. (DOCX)

S1 Table Characteristics of studies validating ICD codes from hospital and death certificate data for stroke and its pathological types. (DOCX)

S2 Table Quality assessment of included studies. (DOCX)

S3 Table Sensitivity of codes for stroke versus a population reference standard. (DOCX)

S4 Table Influence of diagnostic position on PPV. (DOCX)

S5 Table Included primary care Read code studies: characteristics and results. (DOCX)

## References

[1] Lozano R, Naghavi M, Foreman K, Lim S, Shibuya K, Aboyans V, et al Global and regional mortality from 235 causes of death for 20 age groups in 1990 and 2010: a systematic analysis for the Global Burden of Disease Study 2010. The Lancet 2012; 380: 2095–2128. 10.1016/S0140-6736(12)61728-0 PMC10790329 23245604

[2] O’Donnell M, Xavier D, Liu L, Zhang H, Chin SL, Rao-Melacini P, et al Risk factors for ischaemic and intracerebral haemorrhagic stroke in 22 countries (the INTERSTROKE study) a case-controls study. Lancet 2010; 376:112–123. 10.1016/S0140-6736(10)60834-3 20561675

[3] Jackson C, Hutchison A, Dennis M, Wardlaw JM, Lindgren A, Norrivng B, et al Differing risk factor profiles of ischemic stroke subtypes: evidence for a distinct lacunar arteriopathy? Stroke 2010: 41; 624–629. 10.1161/STROKEAHA.109.558809 20150553

[4] Burton P, Hansell A, Fortier I, Manolio TA, Khoury MJ, Little J et al Size matters: just how big is BIG?:quantifying realistic sample size requirements for human genome epidemiology. Int J Epidemiology 2009;38: 263–273. 10.1093/ije/dyn147 PMC2639365 18676414

[5] Giroud M, Lemesle M, Quantin C, Vourch M, Becker F, Milan C, et al A hospital-based and a population-based stroke registry yield different results: the experience in Dijon, France. Neuroepidemiology 1997; 16:15–21. 8994936 10.1159/000109666

[6] Appelros P, Hogeras N, Terent A. Case ascertainment in stroke studies: the risk of selection bias. Acta Neurol Scand 2003;107: 145–149. 12580866 10.1034/j.1600-0404.2003.02120.x

[7] Bejot Y, Mehta Z, Giroud M, Rothwell P. Impact of completeness of ascertainment of minor stroke on stroke incidence: implications for ideal study methods. Stroke 2013:44: 1–7. 10.1161/STROKEAHA.113.000949 23652268

[8] Manuel D, Rosella L, Stukel T. Importance of accurately identifying chronic disease in studies using electronic health records. BMJ 2010: 341: 440–443. 10.1136/bmj.c4226 20724404

[9] Khan N, Harrison S, Rose P. Validity of diagnostic coding within the General Practice Research Database: a systematic review. British Journal of General Practice 2010: e128–e136. 10.3399/bjgp10X483562 20202356 PMC2828861

[10] Andrade S, Harrold L, Tjia J, Cutrona SL, Saczynski JS, Dodd KS, et al A systematic review of validated methods for identifying cerebrovascular accident or transient ischemic attack using administrative data. Pharmacoepidemiology and Drug Safety 2012: 21:100–128. 10.1002/pds.2312 22262598 PMC3412674

[11] Available: http://www.ukbiobank.ac.uk.

[12] Sudlow C, Gallacher J, Allen N, Beral V, Burton P, Danesh J, et al UK Biobank: An Open Access Resource for Identifying the Causes of a Wide Range of Complex Diseases of Middle and Old Age. PLoS Med 2015: 12(3): e1001779 10.1371/journal.pmed.1001779 25826379 PMC4380465

[13] Available: http://www.cdc.gov/nchs/icd.htm.

[14] Available: http://www.icd9data.com/.

[15] Available: http://apps.who.int/classifications/icd10/browse/2010/en.

[16] Hatano S. Experience from a multicentre stroke register: a preliminary report. Bull World Health Organ. 1976; 54: 541–553. 1088404 PMC2366492

[17] Chisholm J. The Read clinical classification. BMJ 1990; 300: 1092 2344534 10.1136/bmj.300.6732.1092 PMC1662793

[18] Stuart-Buttle C, Read J, Sanderson H, Sutton YM. A language of health in action: Read codes, classifications and groupings. Proc AMIA Annu Fall Symp. 1996; 75–79. 8947631 PMC2233183

[19] Ferro JM, Falcao I, Rodrigues G, Ferreira J, Falcao F et al Diagnosis of Transient Ischemic Attack by the non Neurologist. A validation study. Stroke 1996; 27:2225–2229. 8969785 10.1161/01.str.27.12.2225

[20] Albers GW, Caplan LR, Easton JD, Fayad PB, Mohr JP, Saver JL, et al Transient Ischemic Attack—proposal for a new definition. N Engl J Med. 2002;347: 1713–1716. 12444191 10.1056/NEJMsb020987

[21] Brown M, Rudd A, McGovern R. Transient Ischemic Attack—proposed new definition. N Engl J Med. 2003;348: 16. 10.1056/NEJM200304173481621 12700389

[22] Available: http://www.cprd.com/Bibliography/Researchpapers.asp.

[23] Available: http://www.ucl.ac.uk/pcph/research-groups-themes/thin-pub/publications.

[24] Whiting PF, Rutjes A, Westwood M, Mallett S, Deeks J, Reitsma JB, et al QUADAS-2: a revised tool for the quality assessment of diagnostic accuracy studies. Ann Intern Med. 2011;155: 529–536. 10.7326/0003-4819-155-8-201110180-00009 22007046

[25] McCormick N, Lacaille D, Bhole V, Avina-Zubieta JA. Validity of Myocardial Infarction Diagnoses in Administrative Databases: a systematic review. PLoS one. 2014; 9(3): e92286 10.1371/journal.pone.0092286 24682186 PMC3969323

[26] Brown L, Cai T, DasGupta A. Interval estimation for a binomial proportion. Statistical Science 2001; 16: 101–133.

[27] Ruigomez A, Martin-Merino E, Garcia Rodriguez A. Validation of ischemic cerebrovascular diagnoses in the health improvement network (THIN). Pharmacoepidemiology and Drug Safety 2010; 19: 579–585. 10.1002/pds.1919 20131328

[28] Gaist D, Wallander M-A, Gonzalez-Perez A, Garcia-Rodriguez L. Incidence of hemorrhagic stroke in the general population: validation of data from The Health Improvement Network. Pharmacoepidemiology and Drug Safety 2013; 22: 176–182. 10.1002/pds.3391 23229888

[29] Ives D, Fitzpatrick A, Bild D, Psaty B, Kuller L, Crowley PM, et al Surveillance and Ascertainment of Cardiovascular Events. The Cardiovascular Health Study. Ann Epidemiol 1995;5:278–285. 8520709 10.1016/1047-2797(94)00093-9

[30] Lakshminarayan K, Anderson D, Jacobs D, Barber C, Luepker R. Stroke Rates: 1980–2000. The Minnesota Stroke Survey. American Journal of Epidemiology 2009: 169: 1070–1078. 10.1093/aje/kwp029 19318614 PMC2727239

[31] Leibson C, Naessens J, Brown R, Whisnant J. Accuracy of hospital discharge abstracts for identifying stroke. Stroke 1994; 25: 2349–2355. 10.1161/01.str.25.12.2348 7974572

[32] Reker D, Hamilton B, Duncan P, Shu-Chuan J, Rosen A. Stroke: Who’s counting what? Journal of Rehabilitation Research and Development 2001; 38: 281–289. 11392661

[33] Rosamond W, Folsom A, Chambless L, Wang C, McGovern PG, Howard G, et al Stroke Incidence and Survival Among Middle-Aged Adults: 9-year follow-up of the Atherosclerosis Risk in Communities (ARIC) Cohort. Stroke 1999;30: 736–743. 10187871 10.1161/01.str.30.4.736

[34] Roumie C, Mitchel E, Gideon R, Varas-Lorenzo C, Castellsague J, Griffin M. Validation of ICD-9 codes with a high positive predictive value for incident strokes resulting in hospitalization using Medicaid health data. Pharmacoepidemiology and drug safety 2008; 17: 20–26. 17979142 10.1002/pds.1518

[35] Derby C, Lapane K, Feldman H, Carleton R. Trends in Validated cases of fatal and nonfatal stroke, stroke classification, and risk factors in Southeastern New England, 1980 to 1991. Data from the Pawtucket Heart Health Program. Stroke 2000; 31: 875–881. 10753991 10.1161/01.str.31.4.875

[36] Derby C, Lapane K, Feldman H, Carleton R. Possible effect of DRGs on the Classification of Stroke. Implications for Epidemiological Surveillance. Stroke 2001;32:1487–1491. 11441190 10.1161/01.str.32.7.1487

[37] Liu L, Reeder B, Shuaib A, Mazagri R. Validity of Stroke Diagnosis on Hospital Discharge Records in Saskatchewan, Canada: Implications for Stroke Surveillance. Cerebrovascular Dis 1999; 9: 224–230. 10.1159/000015960 10393410

[38] Mayo N, Danys I, Carlton J, Scott S. Accuracy of Hospital discharge coding for stroke. Can J Cardiol 1993; 9: 121D–123D.

[39] Klatsky A, Friedman G, Sidney S, Kipp H, Kubo A, Armstrong M. Risk of Hemorrhagic stroke in Asian American ethnic groups. Neuroepidemiology 2005;25: 26–31. 15855802 10.1159/000085310

[40] Tirschwell D, Longstreth J. Validating Administrative Data in Stroke Research. Stroke 2002; 33: 2465–2470. 12364739 10.1161/01.str.0000032240.28636.bd

[41] Wahl P, Rodgers K, Schneeweiss S, Gage B, Butler J, Wilmer C, et al Validation of claims-based diagnostic and procedure codes for cardiovascular and gastrointestinal serious adverse events in a commercially-insured population. Pharmacoepidemiology and Drug Safety 2010; 19: 596–603. 10.1002/pds.1924 20140892

[42] Goldstein L. Accuracy of ICD-9-CM Coding for the identification of patients with acute ischemic stroke: effect of modifier codes. Stroke 1998; 29: 1602–1604. 9707200 10.1161/01.str.29.8.1602

[43] Benesch C, Witter J, Wilder A, Duncan P, Samsa G, Matchar DB. Inaccuracy of the International Classification of Diseases (ICD-9-CM) in identifying the diagnosis of ischemic cerebrovascular disease. Neurology 1997; 49: 660–664. 9305319 10.1212/wnl.49.3.660

[44] Johnsen S, Overvad K, Sorensen H, Tjonneland A, Husted S. Predictive value of stroke and transient ischemic attack discharge diagnoses in The Danish National Registry of Patients. Journal of Clinical Epidemiology 2002; 55: 602–607. 12063102 10.1016/s0895-4356(02)00391-8

[45] Appelros P, Terent A. Validation of the Swedish Inpatient and cause-of-death registers in the context of stroke. Acta Neurologica Scandinavica 2011; 123: 289–293. 10.1111/j.1600-0404.2010.01402.x 21361878

[46] Krarup L, Boysen G, Janjua H, Prescott E, Truelsen T. Validity of Stroke Diagnoses in a National Register of Patients. Neuroepidemiology 2007; 28: 150–154. 17478969 10.1159/000102143

[47] Tonolen H, Salomaa V, Torppa J, Sivenius J, Immonen-Raiha P, Lehtonen A. The validation of the Finnish Hospital Discharge Register and Causes of Death Register data on stroke diagnoses. European Journal of Cardiovascular Prevention and Rehabilitation 2007;14: 380–385. 17568236 10.1097/01.hjr.0000239466.26132.f2

[48] Ellekjaer H, Holmen J, Kruger O, Terent A. Identification of Incident Stroke in Norway: Hospital Discharge Data Compared With a Population-Based Stroke Register. Stroke 1999;30:56–60. 9880388 10.1161/01.str.30.1.56

[49] Leone M, Capponi A, Varrasi C, Tarletti R, Monaco F. Accuracy of the ICD-9 codes for identifying TIA and stroke in an Italian automated database. Neurol Sci 2004; 25: 281–288. 15624086 10.1007/s10072-004-0355-8

[50] Stegmayr B, Asplund K. Measuring Stroke in the Population: Quality of Routine Statistics in Comparison with a Population Based Stroke Registry. Neuroepidemiology 1992; 11: 204–213. 1291884 10.1159/000110933

[51] Sporalore P, Brocco S, Fedeli U, Visentin C, Schievano E, Avossa F, et al Measuring accuracy of discharge diagnoses for a region-wide surveillance of hospitalized strokes. Stroke 2005; 36: 1031–1034. 15790948 10.1161/01.STR.0000160755.94884.4a

[52] Rinaldi R, Vignatelli L, Galeotti M, Azzimondi G, Carolis P. Accuracy of ICD-9 codes in identifying ischemic stroke in the General Hospital of Lugo di Romagna (Italy). Neurol Sci 2000; 24: 65–69. 10.1007/s100720300074 12827541

[53] Haesebart J, Termoz A, Polazzi S, Mouchoux C, Mechtouff L, Derex L, et al Can Hospital Discharge Databases Be Used to Follow Ischemic Stroke Incidence? Stroke 2013; 44: 1770–1774. 10.1161/STROKEAHA.113.001300 23735951

[54] Aboa-Eboule C, Mengue D, Benzenine E, Hommel M, Giroud M, Bejot Y, et al How accurate is the reporting of stroke in hospital discharge data? A pilot validation study using a population-based stroke registry as control. J Neurol 2013; 260: 605–613. 10.1007/s00415-012-6686-0 23076827 PMC3566387

[55] Palmieri L, Barchielli A, Cesana G, deCampora E, Goldoni C, Sporalore P, et al The Italian Register of Cardiovascular Diseases: Attack Rates and Case Fatality for Cerebrovascular Events. Cerebrovascular Dis 2007; 24: 530–539. 10.1159/000110423 PMC2814017 17971632

[56] Sinha S, Myint P, Luben R, Khaw K-T. Accuracy of death certification and hospital record linkage for identification of incident stroke. BMC Medical Research Methodology 2008; 8: 74 10.1186/1471-2288-8-74 19000303 PMC2605452

[57] Wright L, Green J, Canoy D, Carins B, Balkwill A, Beral V. Vascular disease in women: comparison of diagnoses in hospital episode statistics and general practice records in England. BMC Medical Research Methodology 2012; 12: 161 10.1186/1471-2288-12-161 23110714 PMC3514155

[58] Davenport R, Dennis M, Warlow C. The Accuracy of Scottish Morbidity Record (SMR1) Data for Identifying Hospitalised Stroke Patients. Health Bulletin 1996; 54: 402–405. 8936808

[59] Mant J, Mant F, Winner S. How good is routine information? Validation of coding for acute stroke in Oxford hospitals. Health Trends 1997;29: 96–99.

[60] Barer D, Ellul J. Correcting outcome data for case mix in stroke medicine. BMJ 1996;313: 1005–1006. 8892433 10.1136/bmj.313.7063.1005c PMC2352292

[61] Panayiotou B, Fotherby M, Potter J, Castleden C. The accuracy of diagnostic coding of cerebrovascular disease. Medical Audit News 1993; 3: 153–155.

[62] Hasan M, Meara R, Bhowmick B. The Quality of Diagnostic Coding in Cerebrovascular Disease. International Journal for Quality in Health Care 1995; 7: 407–410. 8820217 10.1093/intqhc/7.4.407

[63] Kirkman M, Mahattanakul W, Gregson B, Mendelow D. The Accuracy of Hospital Discharge Coding for Hemorrhagic Stroke. Acta Neurol Belg 2009; 109: 114–119. 19681442

[64] Koster M, Asplund K, Johansson A, Stegmayr B. Refinement of Swedish Administrative Registers to Monitor Stroke events on the National Level. Neuroepidemiology 2013; 40: 240–246. 10.1159/000345953 23364278

[65] Harriss L, Ajani A, Hunt D, Shaw J, Chambers B, Dewey H, et al Accuracy of national mortality codes in identifying adjudicated cardiovascular deaths. Australian and New Zealand Journal of Public Health 2011;35: 466–76. 10.1111/j.1753-6405.2011.00739.x 21973254

[66] Mant J, McManus R, Hare R, Mayer P. Identification of stroke in the community: a comparison of three methods. British Journal of General Practice 2003;53:520–524. 14694663 PMC1314641

[67] Tu K, Wang M, Young J, Green D, Ivers N, Butt D et al Validity of administrative data for identifying patients who have had a stroke or transient ischemic attack using EMRALD as a reference standard. Canadian Journal of Cardiology 2013; 29: 1388–1394. 10.1016/j.cjca.2013.07.676 24075778

[68] Flynn R, Macdonald T, Schembri N, Murray G, Doney A. Automated data capture from free-text radiology reports to enhance accuracy of hospital inpatient stroke codes. Pharmacoepidemiology and drug safety. 2010; 19:843–847. 10.1002/pds.1981 20602346

[69] Thiru K, Hassey A, Sullivan F. Systematic review of scope and quality of electronic patient record data in primary care. BMJ 2003; 326:1070 12750210 10.1136/bmj.326.7398.1070 PMC155692

[70] Herrett E, Thomas S, Schoonen W, Smeeth L, Hall A. Validation and validity of diagnoses in the General Practice Research Database: a systematic review. Br J Clin Pharmacol 2010; 69: 4–14. 10.1111/j.1365-2125.2009.03537.x 20078607 PMC2805870

[71] Burns E, Rigby E, Mamidanna R, Bottle A, Aylin P, Ziprin P, Faiz O. Systematic review of discharge coding accuracy. Journal of Public Health 2011; 34: 138–148. 10.1093/pubmed/fdr054 21795302 PMC3285117

[72] Jordan K, Porcheret M, Croft P. Quality of morbidity coding in general practice computerized medical records: a systematic review. Family Practice 2004; 21: 396–412. 15249528 10.1093/fampra/cmh409

[73] Campbell SE, Campbell MK, Grimshaw JM, Walker AE. A systematic review of discharge coding accuracy. Journal of Public Health Medicine 2001; 23: 205–211. 11585193 10.1093/pubmed/23.3.205

[74] Kessler C, Freyberger HJ, Dittman V, Ringelstein EB. Interrater reliability in the assessment of neurovascular diseases. Cerebrovascular diseases 1991; 1: 43–48.

[75] Dixon J, Sanderson C, Elliot P, Walls P, Jones J, Petticrew M. Assessment of the reproducibility of clinical coding in routinely collected hospital activity data: a study in two hospitals. Journal of Public Health Medicine 1998;20: 63–69. 9602451 10.1093/oxfordjournals.pubmed.a024721

[76] Ferro JM, Falcao I, Rodrigues G, Canhao P, Melo TP, Falcao F, et al Diagnosis of Transient Ischaemic Attack by the non-neurologist. A validation study. Stroke. 1996;27: 2225–2229. 8969785 10.1161/01.str.27.12.2225

[77] Wardlaw JM, Mielke M. Early Signs of Brain Infarction at CT: Observer Reliability and Outcome after Thrombolytic Treatment—Systematic Review. Radiology. 2005; 235: 444–453. 15858087 10.1148/radiol.2352040262

[78] Fiebach JB, Schellinger PD, Jansen O, Meyer M, Wilde P, Bender J, et al CT and Diffusion Weighted-MR imaging in randomized order. Diffusion-Weighted Imaging results in higher accuracy and lower inter-rater variability in the diagnosis of hyperacute ischemic stroke. Stroke. 2002;33: 2206–2210. 12215588 10.1161/01.str.0000026864.20339.cb

[79] Bamford J, Sandercock P, Warlow C, Gray M. Why are patients with acute stroke admitted to hospital? BMJ 1986; 292: 1369 3085852 10.1136/bmj.292.6532.1369 PMC1340376

[80] Rothwell PM, Coull AJ, Giles MF, Howard SC, Silver LE, Bull LM, et al Change in stroke incidence, mortality, case-fatality, severity, and risk factors in Oxfordshire, UK from 1981 to 2004 (Oxford Vascular Study). The Lancet 2004; 363: 1925–1933. 10.1016/S0140-6736(04)16405-2 15194251

[81] Schulz U, Rothwell P. Differences in Vascular Risk Factors between etiological subtypes of ischemic stroke: importance of population-based studies. Stroke 2003;34:2050–2059. 12829866 10.1161/01.STR.0000079818.08343.8C

[82] Van Staa T-P, Abenhaim L. The Quality of Information Recorded on a UK Database of Primary Care Records: A Study of Hospitalizations due to Hypoglycaemia and Other Conditions. Pharmacoepidemiology and Drug Safety 1994; 3: 15–21.

[83] Available: http://www.isdscotland.org/.

[84] Available: http://www.saildatabank.com/.

