# Supplementary material for: Accuracy of Electronic Health Record Data for Identifying Stroke Cases in Large-Scale Epidemiological Studies: A Systematic Review from the UK Biobank Stroke Outcomes Group
Source: PLoS One. 2015 Oct 23;10(10):e0140533. doi: 10.1371/journal.pone.0140533 (PMC4619732; doi:10.1371/journal.pone.0140533)
Supplement: S1 Appendix — (DOCX) [file pone.0140533.s001.docx]

**S1 Appendix. Study protocol**

**Accuracy of electronic health record data for identifying stroke cases in large-scale epidemiological studies: a systematic review from the UK Biobank stroke outcomes group.**

Rebecca M Woodfield, MA MRCP(E), Ian Grant, PhD, UK Biobank Stroke Outcomes Group, UK Biobank Follow-up and Outcomes Working Group, Cathie LM Sudlow, DPhil FRCP(E)

**1. Review Question(s)**

1.1 Primary Question

Accuracy (positive predictive value) of coded health record data (International Classification of Diseases codes, ICD, or Read codes) for stroke and its main pathological types (ischaemic stroke, intra-cerebral haemorrhage, subarachnoid haemorrhage) using WHO or equivalent definitions in an adult population.

1.2 Secondary Questions

A. Sensitivity, specificity, and NPV (negative predictive value) of coded health record data for stroke and its main pathological types (amongst studies where the reference standard is population-based).

B. Influence of the range of codes selected (amongst ICD and/or Read codes for ‘cerebrovascular disease’) on PPV and/or sensitivity for stroke and its main types.

C. Influence of diagnostic position (primary versus secondary) on the PPV and/or sensitivity of ICD codes for stroke and its main pathological types.

D. Accuracy (PPV, sensitivity, specificity, NPV) of multiple sources of coded data (ICD codes from death certificates, and/or ICD codes from hospital records, and/or Read codes from primary care) for stroke and its main pathological types.

**2. Searches**

We will search the following databases from 1990 to the date of search:

MEDLINE (Ovid SP);

EMBASE (Ovid SP)

Clinical Research Practice Datalink

The Health Improvement Network

We will review bibliographies of included publications for any additional relevant articles.

One author will review all titles and abstracts (IG). A second author (RW) will review a 10% sample of titles and abstracts. Two authors will independently review all potentially relevant full texts and select studies for inclusion (IG and RW). Any areas of uncertainty will be discussed and resolved with a third (CS).

2.1 MEDLINE search strategy

1. (cerebrovascular disorders/ or exp basal ganglia cerebrovascular disease/ or exp brain ischemia/ or exp carotid artery diseases/ or exp cerebral small vessel diseases/ or exp intracranial arterial diseases/ or exp intracranial embolism/ and thrombosis/) or exp intracranial hemorrhages/ or stroke/ or exp brain infarction/ or stroke, lacunar/ or vertebral artery dissection/

2. (stroke or cerebrovasc$ or brain vasc$ or cerebral vasc$ or cva$ or appoplex$ or isch?emi$ attack$ or tia$ or SAH).tw.

3. ((brain$ or cerebr$ or cerebell$ or cortical or vertebrobasilar or hemisphere$ or intracran$ or intracerebral or infratentorial or supratentorial or MCA or anterior circulation or posterior circulation or basal ganglia) adj5 (isch?emi$ or infarct$ or thrombo$ or emboli$)).tw.

4. ((brain$ or cerebr$ or cerebell$ or intracerebral or intracran$ or parenchymal or intraventricular or infratentorial or supratentorial or basal gangli$ or subarachnoid or putaminal or putamen or posterior fossa) adj5 (haemorrhage$ or hemorrhage$ or haematoma$ or hematoma$ or bleed$)).tw.

5. 1 or 2 or 3 or 4

6. medical record/ or medical record review/ or Medical Records Systems, Computerized/ or medical information system/

7. (hospital or GP or medical or general practitioner or health).tw.

8. international classification of diseases/ or Disease/cl [Classification]

9. Clinical Coding/

10. read coding.tw.

11. (ICD-10 or ICD-9 or ICD-9-CM or ICD-10-CM).tw.

12. 6 or 7 or 8 or 9 or 10 or 11

13. exp Sensitivity/ and Specificity/

14. exp Validation Studies/ or exp predictive value/ or exp Reproducibility of Results/

15. (sensitivity or specificity or positive predictive value).tw.

16. (validity or reproducibility).tw.

17. 13 or 14 or 15 or 16

18. 12 and 17

19. 5 and 18

2.2 EMBASE search strategy

1. cerebrovascular disease/ or basal ganglion hemorrhage/ or exp brain hematoma/ or exp brain hemorrhage/ or exp brain infarction/ or exp brain ischemia/ or exp carotid artery disease/ or cerebal artery disease/ or cerebrovascular accident/ or exp occlusive cerebrovascular disease/ or exp stroke/

2. stroke unit/ or stroke patient.mp. [mp=title, abstract, subject headings, heading word, drug trade name, original title, device manufacturer, drug manufacturer, device trade name, keyword]

3. (stroke or cerebrovasc$ or brain vasc$ or cerebral vasc$ or cva$ or apoplexy$ or isch?emi$ attack$ or tia$ or SAH).tw.

4. ((brain$ or cerebr$ or cerebell$ or cortical or vertebrobasilar or hemisphere$ or intracran$ or intracerebral or infratentorial or supratentorial or MCA or anterior circulation or posterior circulation or basal ganglia) adj5 (isch?emi$ or infarct$ or thrombo$ or emboli$)).tw.

5. ((brain$ or cerebr$ or cerebell$ or intracerebral or intracran$ or parenchymal or intraventricular or infratentorial or supratentorial or basal gangli$ or subarachnoid or putaminal or putamen or posterior fossa) adj5 (haemorrhage$ or hemorrhage$ or haematoma$ or hematoma$ or bleed$)).tw.

6. 1 or 2 or 3 or 4 or 5

7. medical record/ or medical record review/ or electronic medical record/ or medical information system/

8. (hospital or GP or medical or general practitioner or health).tw.

9. "international classification of diseases"/ or disease classification/

10. coding/ or "read coding"/

11. read coding.tw.

12. (ICD-10 or ICD-9 or ICD-9-CM or ICD-10-CM).tw.

13. 7 or 8 or 9 or 10 or 11 or 12

14. "sensitivity and specificity"/

15. exp validation study/ or exp predictive value/ or exp reproducibility/

16. (sensitivity or specificity or positive predictive value).tw.

17. (validity or reproducibility).tw.

18. 14 or 15 or 16 or 17

19. 13 and 18

20. 5 and 19

**3. Types of study to be included**

We will include studies in adult populations (cohort studies, case-control studies, or clinical trials) which compare ICD codes or Read codes for ‘cerebrovascular disease’ against a second source of data for stroke using WHO, or equivalent definitions.

We will exclude studies in highly selected populations with an increased risk of stroke (due to the influence of prevalence on PPV).

Studies are required to report the group(s) of codes validated and the Positive Predictive Value (or data from which this can be calculated).

Studies should use a reference standard of ‘stroke’ (distinguished from transient ischaemic attack or generalised cerebrovascular disease) when calculating PPV, sensitivity, specificity, and NPV.

We will exclude studies which assess < 50 coded events (due to limited precision).

3.1 Condition or domain being studied

International Classification of Diseases codes (version 9 or any later version) for cerebrovascular diseases based on hospital admission or death certificate diagnoses.

Read codes (any version) for cerebrovascular diseases.

Studies should publish the group of codes validated (so that the influence of individual code selection on accuracy can be explored).

We will exclude studies of ICD version 8, or earlier primary care systems (e.g., OXMIS codes) because these have been superseded by newer coding systems in the UK.

3.2 Participants/population

Any adult population.

We will not exclude studies based on participant selection criteria (e.g., age, cardiovascular risk, education, cognitive impairment, disability).

We have specified secondary questions which will examine the influence of participant characteristics (e.g., age and stroke prevalence) on the accuracy of coded data for stroke/main stroke types.

3.3 Interventions, exposures

***Reference standard***: We accept that the inter-observer reliability of stroke diagnosis is imperfect, even amongst experts. In the absence of a true ‘gold standard’ for stroke, we will include studies which use any of the following reference standards for stroke: clinical examination; physician questionnaire; medical record review (primary care and/or hospital records); stroke registers (informed by multiple overlapping data sources, ‘hot pursuit’, and expert medical record review).

Studies should use a clinical syndrome-based definition (WHO, or equivalent) for diagnosing stroke.

3.4 Comparators/control

Not applicable

**4. Context**

The principle criterion is accuracy of coded data (ICD or Read codes for cerebrovascular diseases) for the ascertainment (sensitivity) and confirmation (PPV) of stroke cases in relatively unselected adult populations.

We have pre-specified questions to see if PPV and/or sensitivity for stroke and/or its main types are influenced by the groups of cerebrovascular diseases codes selected, or the diagnostic position of these codes (primary versus primary or secondary).

We will exclude studies which include subdural haemorrhage, unspecified cerebrovascular disease and/or TIA in their reference standard definition of stroke.

**5. Outcomes**

5.1 Primary outcomes

We will calculate Positive predictive value (PPV) of coded data for stroke and/or its main types (ischaemic stroke, intracerebral haemorrhage, subarachnoid haemorrhage) in all included studies using the available published data.

5.2 Secondary outcomes

In studies which use population-based reference standards, we will calculate sensitivity, specificity, PPV, NPV and stroke prevalence using 2x2 contingency tables.

The reference standard will be grouped into hospital-based versus population-based according to the following definitions:

Population-based: primary care medical records and/or general practitioner questionnaires and/or population-based stroke registers used to capture strokes diagnosed out of hospital.

Hospital-based: hospital medical records only +/- hospital physician questionnaires which largely capture hospitalised strokes.

If an individual study uses more than one group of cerebrovascular disease codes, more than one diagnostic position (primary versus primary or secondary), or more than one data source (hospital versus death certificate codes versus both), we will explore the influence of code selection, diagnostic position, and code source on PPV/sensitivity for stroke and its main types.

Calculations will be performed independently by two authors (IG and RW) using available published data. Disagreements will be resolved through discussion with a third (CS).

**6. Data extraction (selection and coding)**

We will extract data onto study-specific proforma.

6.1 Covariates of interest:

- Study author

- Publication date

- Country

- Target population age (range)

- Range of ICD/Read codes validated

- Code version (eg. ICD-9, ICD-9-CM, ICD-10)

- Code source (hospital, death certificate or both)

- Number of coded events assessed

- Diagnostic position of codes (primary, secondary or both)

- Diagnosis sought (eg. stroke, ischaemic stroke, haemorrhagic stroke, intracerebral haemorrhage, subarachnoid haemorrhage, or any combination).

- Reference standard used (eg expert hospital record review, population-based stroke register)

- Number of coded events confirmed (depending on the diagnosis sought, eg., stroke or one of its main types)

- PPV of selected codes for stroke and/or its main types

- Sensitivity of selected codes for stroke and/or its main types (when the reference standard is population-based).

6.2 Study-level quality assessment

We will assess methodological quality using a modification of the Quality Assessment of Diagnostic Accuracy Studies tool (QUADAS-2). QUADAS-2 uses fourteen questions to assess study-level risk of bias and generalisability to the target population.

QUADAS-2 was recently modified for a systematic review of the validity of Myocardial Infarction Diagnoses in Administrative Databases. [McCormick et al. PLoS ONE 2014 9 (3) e92286.]. This new version had fewer questions for the assessment of bias and additional questions for the assessment of reporting quality. We will use these questions (because they were developed specifically for studies of the accuracy of coded data), removing the question ‘were the index test results interpreted without knowledge of the results of the reference standard’ because we feel that the codes (index test) are unlikely to be misinterpreted, and including the question ‘was the study UK-based (?)’ to assess generalisability to the UK population.

Our modified QUADAS-2 will therefore include five questions for the assessment of reporting quality, three for assessment of generalisability, and six for assessment of risk of bias (fourteen in total). Each question will score ‘low’, ‘high’, or ‘unclear’ reporting quality/generalisability/risk of bias according to the rules (below).

An overall quality score (0-14) will be derived for each study by adding the number of questions which scored ‘high reporting quality’, ‘high generalisability’, and ‘low risk of bias’.

- 1. Modified QUADAS-2 for assessment of study quality:

1. ***Assessment of reporting quality***
2. ***Were the selection criteria clearly described?***

High reporting quality: population selection criteria clearly described.

Low reporting quality: population selection criteria unclear.

1. ***Was execution of the index test described in sufficient detail to allow replication of the test? (The index test was the coding algorithm used in the study.)***

Studies were excluded from this review if the codes used were not reported.

High reporting quality: diagnostic position of codes (primary vs. primary or secondary diagnoses) clearly reported.

Low reporting quality: diagnostic position of codes not clearly reported.

1. ***Was execution of the reference standard described in sufficient detail to permit its replication?***

High reporting quality: reported the information used to make the reference standard diagnosis (eg., full medical record vs. abstracted data, brain imaging to confirm stroke subtypes) and described the adjudicators’ expertise (eg., expert stroke physician).

Low reporting quality: did not report the information used to make the reference standard diagnosis and/or did not report adjudicators’ expertise.

1. ***If participants were excluded from the final analysis, were they described and were the reasons for this exclusion explained?***

High reporting quality: reported the numbers excluded from the final analyses and the reason(s), or there were no exclusions.

Low reporting quality: did not report the numbers excluded from the final analyses and the reason(s).

1. ***Were uninterpretable/intermediate results reported?***

High reporting quality: ‘uncertain’ diagnoses were reported (eg., if there was ‘insufficient information in the medical record to make a diagnosis, and/or it was reported if ‘possible strokes’ were included in the ‘confirmed stroke’ category.)

Low reporting quality: no reporting of ‘uncertain’ results.

1. ***Assessment of generalisability (to UK population)***
2. ***Country: was the study UK-based?***

High generalisability: UK based population.

Low generalisability: non-UK based population.

1. ***Population selected: was the spectrum of patients selected representative of the patients who will receive the diagnosis in practice?***

High generalisability: the study included patients diagnosed and treated in a representative mixture of specialist and non-specialist settings, and the population was otherwise relatively unselected.

Low generalisability: the study was performed in a more selected population (eg., restricted to patients admitted to a specialist stroke unit, where coding performance might be higher).

Unclear: insufficient published information.

1. ***Reference standard: were the same clinical data available when test results were interpreted as would be available when used in practice?***

High generalisability: medical record data (extracts or full record) including brain imaging data (original scans/written reports). Brain imaging would be used in current practice to exclude stroke mimics/diagnose stroke subtypes.

Low generalisability: brain imaging (original scans/reports) not available.

Unclear generalisability: insufficient published information.

***3) Assessment of risk of bias***

***a)*** ***Selection bias: did the whole sample, or a random selection of the sample, receive verification using a reference standard of diagnosis?***

Low risk of bias: The whole sample/random selection of the sample received verification using medical records/physician questionnaire.*

High risk of bias: some of the sample did not receive verification because reference standard data were missing* (missing records/unreturned questionnaires).

Unclear risk of bias: insufficient information published.

*It is assumed that if the reference standard was a prospectively generated, expert-led, population-based stroke register, which used multiple sources of case ascertainment and confirmation, that this reference standard is ‘complete’ (ie., it is unlikely to have missed ‘true positive’ stroke cases).

***b) Blinding:*** ***were the reference standard results interpreted without knowledge of the results of the index test?***

Low risk of bias: blinding present, or the reference standard diagnosis was made prior to the study (eg., the reference standard was a prospectively generated stroke register)

High risk of bias: blinding not present, or not reported.

***c) Independence: was the reference standard independent of the index test?***

Low risk of bias: the reference standard was independent of the index test.

High risk of bias: the index test formed part of the reference standard (eg., coded diagnoses were used to identify stroke cases for a population based register, and there was no further confirmation).

***d) Differential verification: did all patients receive the same reference standard regardless of the index test result?***

Low risk of bias: yes

High risk of bias: some/all code positive cases received different reference standards from code negative cases eg., stroke code positive cases not present in a stroke register (potential false positive cases) were selected for subsequent medical record review, but stroke code negative cases present in stroke register (potential false negative cases) did not have subsequent medical record review.

***e) Reference standard: is the reference standard likely to correctly classify the target condition?***

Low risk of bias: the reference standard was likely to identify all hospital admitted strokes (plus strokes in the community for death certificate codes), AND, was either based on an expert (neurologist or stroke physician) reviewing the full medical record, or was based on a non-expert (eg., research assistant, research nurse, or ‘adjudicator’) following clearly described rules (eg., using CT reports to exclude stroke mimics or using CT reports to differentiate haemorrhage from ischaemic stroke, where applicable).

High risk of bias: the study validated death certificate codes and the reference standard was not population-based (and therefore risked misclassifying true strokes diagnosed out of hospital as false positive codes), and/or the diagnosis was made by a non-expert and there was not a clear protocol to exclude stroke mimics or to differentiate haemorrhagic from ischaemic stroke

Unclear: insufficient published data.

***f) Timing: was the time period between the reference standard and the index test short enough to be reasonably sure that the target condition did not change between the two tests?***

Low risk of bias: The information used to make the reference standard diagnosis was the same as the information used at the time of coding.

High risk of bias: The information used to make the reference standard diagnosis was not the same as the information used at the time of coding.

Unclear: insufficient published data.

**7. Strategy for data synthesis**

We will cross classify coded stroke diagnoses with the reference standard diagnosis (‘stroke’ versus ‘non-stroke’).

PPV (%) = true positive codes / [true positive codes + false positive codes] x 100

NPV (%) = true negative codes / [true negative codes + false negative codes] x 100

True positive codes = coded diagnosis ‘stroke’ and reference standard ‘stroke’.

False positive codes = coded diagnosis ‘stroke’ and reference standard ‘non-stroke’.

True negative codes = coded diagnosis ‘non-stroke’ and reference standard ‘non-stroke’.

False negative codes= coded diagnosis ‘non-stroke’ and reference standard ‘stroke’.

Where the reference standard is population based, we will construct standard 2x2 tables describing binary test results (coded diagnosis ‘stroke’ and coded diagnosis ‘non-stroke’) cross classified with binary reference standard results (‘stroke’ and ‘non-stroke’).

We will use this data to calculate sensitivity, specificity, PPV, NPV, and 95% confidence intervals.

We will tabulate results for visual inspection to assess the influence of individual code selection, diagnostic position, and code source on the PPV, sensitivity, specificity and NPV of coded data for stroke/its main types. Where possible, and to limit the impact of between-study heterogeneity, we will use within-study as well as between-study comparisons.

We will assess heterogeneity between studies by inspection of tabulated data.

We will not quantify publication bias as there is no assessment applicable to test accuracy.

**8. Dissemination plans**

We will present our findings at local, national and international meetings. We plan to publish a full paper in a peer-reviewed scientific journal.
