# Supplementary material for: Accuracy of Electronic Health Record Data for Identifying Stroke Cases in Large-Scale Epidemiological Studies: A Systematic Review from the UK Biobank Stroke Outcomes Group
Source: PLoS One. 2015 Oct 23;10(10):e0140533. doi: 10.1371/journal.pone.0140533 (PMC4619732; doi:10.1371/journal.pone.0140533)
Supplement: S1 Table — (DOCX) [file pone.0140533.s002.docx]

**S1 Table. Characteristics of studies validating ICD codes from hospital and death certificate data for stroke and its pathological types.^*^**

| Study | | Country | | Age (range) | ICD code group | | ICD version | | Code  source | Coded events assessed (n)^†^  (a) | | Diagnostic position | Reference standard^‡^ | Coded events confirmed (n)  (b) | | PPV  (% & 95% CI)  (b/a) | Score^§^  (14) |
| --- | --- | --- | --- | --- | --- | --- | --- | --- | --- | --- | --- | --- | --- | --- | --- | --- | --- |
| **Studies validating codes for stroke** | | | | | | | | | | | | | | | | |  |
| Mayo  1993 | | Canada | | - | 430-434, 436, 437 | | | 9 | H | | 96 | P | Medical Record | 72 | 76 (67 to 83) | | 8 |
| Liu  1999 | | Canada | | - | 430-438 | | | 9 | H | | 862 | P/S | Medical Record^¶^ | 487 | 56 (53 to 60) | | 8 |
|  |  |  |  |  | 430-438 | | |  |  | | 621 | P |  | 417 | 67 (63 to 71) | |  |
|  |  |  |  |  | 430-438 | | |  |  | | 327 | P/S |  | 151 | 46 (41 to 52) | |  |
|  |  |  |  |  | 430-438 | | |  |  | | 213 | P |  | 129 | 61 (54 to 67) | |  |
| Leibson  1994 | | US | | - | 430-438 | | | 9 | H | | 377 | P | Population Register | 225 | 60 (55 to 65) | | 11 |
|  |  |  |  |  | 430-438 | | |  |  | | 462 | P/S |  | 249 | 54 (49 to 58) | |  |
|  |  |  |  |  | 430-438 | | |  |  | | - | P | Hospital Register | 239 | - | |  |
|  |  |  |  |  | 430-438 | | |  |  | | - | P/S |  | 290 | - | |  |
| Rosamond  1999 | | US | | 45-64 | 430-438 | | | 9-CM | H | | 1058 | - | Medical Record^**^ | 326 | 31 (28 to 34) | | 12 |
|  |  |  |  |  | 430-434 | | |  |  | | 526 |  |  | 234 | 44 (40 to 49) | |  |
| Reker  2001 | | US | | - | 430-438 | | | 9-CM | H | | 671 | - | Medical Record^**^ | 279 | 42 (38 to 45) | | 6 |
|  |  |  |  |  | 430, 431, 433, 434, 436 | | |  |  | | 334 | - |  | 198 | 59 (54 to 64) | |  |
|  |  |  |  |  | 430, 431, 432, 434, 436 OR  430-438^††^ | | |  |  | | 491 | P/S | Inpatient register | 254 | 52 (47 to 56) | |  |
|  |  |  |  |  | 431.x, 433.x1, 434.x1 | | |  |  | | 200 | P/S |  | 150 | 75 (69 to 80) | |  |
| Klatsky  2005 | | US | | - | 430-438 | | | 9 | H | | 3239 | P | Medical Record^**^ | 2494 | 77 (76 to 78) | | 10 |
| Leone  2004 | | Italy | | - | 430-438 | | | 9 | H | | 1017 | P/S | Inpatient Register | 609 | 60 (57 to 63) | | 11 |
|  |  |  |  |  | 430-438 | | |  |  | | 833 | P |  | 550 | 66 (63 to 69) | |  |
|  |  |  |  |  | 430, 431, 434, 436 | | |  |  | | 411 | P/S |  | 371 | 90 (87 to 93) | |  |
|  |  |  |  |  | 430, 431, 434, 436 | | |  |  | | 375 | P |  | 353 | 94 (91 to 96) | |  |
| Sporalore  2005 | | Italy | | - | 430-434, 436-438 | | | 9 | H | | 3619 | P/S | Medical Record | 1296 | 36 (34 to 37) | | 9 |
|  |  |  |  |  | 430-434, 436-438 | | |  |  | | 2174 | P |  | 1021 | 47^‡‡^ (45 to 49) | |  |
| Palmieri  2007 | | Italy | |  | 342, 430-438 | | | 9 | H+D | | 2793 | P/S | Population register | 1173 | 42 (40 to 44) | | 8 |
| Stegmayr  1992 | | Sweden | | 25-74 | 430-438 | | | 9 | D | | 899 | P/S | Population Register | 812 | 90 (88 to 92) | | 12 |
|  |  |  |  |  | 430-438 | | |  | H | | 5101 | P/S |  | 3492 | 69 (67 to 70) | |  |
| Ellekjaer  1999 | | Norway | | ≥ 15 | 430-438 | | | 9 | H | | 759 | P/S | Population Register | 369 | 49 (45 to 52) | | 9 |
|  |  |  |  |  | 430,431,434, 436 | | |  |  | | 508 | P/S |  | 347 | 68 (64 to 72) | |  |
| Panayiotou  1993 | | UK | | 25-100 | 430-438 | | | 9 | H | | 117 | P | Inpatient Register | 94 | 80 (72 to 87) | | 8 |
| Hasan  1996 | | UK | | 60-94 | 430-438 | | | 9 | H | | 166 | P | Medical Record | 113 | 68 (61 to 75) | | 9 |
| Harriss  2010 | | Australia | | 40-69 | 430-438, I60-I69 | | | 9 +10 | D | | 119 | P | Medical Record | 72 | 61 (52 to 69) | | 12 |
|  |  |  |  |  | 430-431, 433-434, I60, I61, I63, I690, I691, I693 | | |  |  | | 61 | P |  | 54 | 89 (78 to 94) | |  |
|  |  |  |  |  | 430-438, I60-I69 | | |  |  | | - | P/S |  | - | - | |  |
| Johnsen  2002 | | Denmark | | 50-64 | I60-I69 and G45 | | | 10 | H | | 565 | P/S | Medical Record | 325 | 58 | | 10 |
|  |  |  |  |  | I60, I61, I63, I64 | | |  |  | | 378 | P/S |  | 299 | 79 (75 to 83) | |  |
| Krarup  2007 | | Denmark | | - | I60-I69, G45 | | | 10 | H | | 236 | - | Medical Record | 153^‡‡^ | 65^‡‡^ (59 to 71) | | 9 |
|  |  |  |  |  | I60, I61, I63, I64 | | |  |  | | 164 | - |  | 136^‡‡^ | 83^‡‡^ (76 to 88) | |  |
| Sinha  2008 | | UK | | 40-79 | I60-I69 | | | 10 | H + D | | 250 | P/S | Medical Record | 191 | 76 (71 to 81) | | 11 |
| Roumie  2008 | | US | | 50-84 | 430, 431, 433.x1, 434.x1, 436^§§^ | | | 9-CM | H | | 231 | P/S | Medical Record^**^ | 205 | 89 (84 to 92) | | 9 |
|  |  |  |  |  | 430, 431, 433.x1, 434.x1, 436 | | | 9-CM |  | | 203 | P | Medical Record^**^ | 196 | 97 (93 to 98) | |  |
| **Derby*  *2000* | | *US* | | *35-74* | *431, 432, 434, 435, 436, 437* | | | *9* | *H* | | *3811* | *P/S* | *Medical Record*^***^ | *2269* | *60 (58 to 61)* | | *11* |
| **Derby*  *2001* | | *US* | | *35-74* | *431, 432, 434, 436, 437* | | | *9* | *H* | | *2124* | *P* | *Medical Record*^***^ | *1699* | *80 (78 to 82)* | | *11* |
| **Lakshminarayan*  *2009* | | *US* | | *30-74* | *431, 432, 434, 436, 437* | | | *9* | *H* | | *6032* | *P/S* | *Medical Record* | *3773* | *63 (61 to 64)* | | *9* |
|  |  |  |  |  | *431, 432, 434, 436, 437* | | |  |  | | *4445* | *P* |  |  | *85 (84 to 86)* | |  |
| **Davenport*  *1996* | | *UK* | | *≥ 18* | *431, 433-438* | | | *9* | *H* | | *557* | *P* | *Inpatient Register* | *529* | *95 (93 to 96)* | | *11* |
| **Barer*  *1996* | | *UK* | | *-* | *431, 433, 434, 436* | | | *9* | *H* | | *340* | *-* | *Hospital Register* | *278* | *82 (77 to 86)* | | *8* |
| **Mant*  *1998* | | *UK* | | *-* | *431, 432.9, 434, 436, 437.0, 437.1, 437.9* | | | *9* | *H* | | *318* | *P/S* | *Inpatient Register* | *230* | *72 (67 to 77)* | | *11* |
| **Ives*  *1995* | | *US* | | *≥ 65* | *430, 431, 432.9, 434, 436* | | | *9-CM* | *H* | | *79* | *-* | *Medical Record* | *71* | *90 (81 to 95)* | | *8* |
| **Appelros*  *2011* | | *Sweden* | | *-* | *I61, I63, I64* | | | *10* | *D* | | *98* | *-* | *Population Register* | *78* | *80 (71 to 86)* | | *11* |
|  |  |  |  |  | *I61, I63, I64* | | |  | *H* | | *328* | *-* |  | *318* | *97 (94 to 98)* | |  |
|  |  |  |  |  | *I61, I63, I64* | | |  | *H + D* | | *363* | *-* |  | *333* | *92 (88 to 94)* | |  |
| **Koster*  *2013* | | *Sweden* | | *≥ 20* | *I61, I63, I64* | | | *10* | *D* | | *102* | *P/S* | *Population Register* | *40* | *39 (30 to 49)* | | *12* |
|  |  |  |  |  |  |  |  |  | *H* | | *1426* | *P/S* |  | *1224* | *86 (84 to 88)* | |  |
|  |  |  |  |  |  |  |  |  | *H + D* | | *1526* | *P/S* |  | *1264* | *83 (81 to 85)* | |  |
| **Aboa-Eboule*  *2013* | | *France* | | *-* | *I61, I63, I64, G46* | | | *10* | *H* | | *903* | *P* | *Hospital Register* | *625* | *69 (66 to 72)* | | *11* |
| **Studies validating codes for ischemic stroke** | | | | | | | | | | | | | | | | |  |
| Benesch  1997 | US | | - | | 433, 434, 436 | | 9-CM | | H | | 550 | P/S | Medical Record | 234 | 43 (38 to 47) | | 4 |
|  |  |  |  |  | 433, 434, 436 | |  |  |  | | 379 | P |  | 199 | 53 (47 to 57) | |  |
|  |  |  |  |  | 434, 436 | |  |  |  | | 250 | P/S |  | 216 | 86 (82 to 90) | |  |
|  |  |  |  |  | 434, 436 | |  |  |  | | 203 | P |  | 183 | 90 (85 to 94) | |  |
|  |  |  |  |  | 433, 434 | |  |  |  | | 521 | P/S |  | 210 | 40 (36 to 45) | |  |
|  |  |  |  |  | 433, 434 | |  |  |  | | 361 | P |  | 183 | 51 (46 to 56) | |  |
|  |  |  |  |  | 433 | |  |  |  | | 295 | P/S |  | 18 | 6 (4 to 9) | |  |
|  |  |  |  |  | 433 | |  |  |  | | 176 | P |  | 16 | 9 (6 to 14) | |  |
|  |  |  |  |  | 434 | |  |  |  | | 226 | P/S |  | 192 | 85 (80 to 89) | |  |
|  |  |  |  |  | 434 | |  |  |  | | 185 | P |  | 167 | 91 (85 to 94) | |  |
| Goldstein  1998 | US | | - | | 434 | | 9-CM | | H | | 108 | P | Discharge summary^***^ | 88 | 82 (73 to 88) | | 7 |
|  |  |  |  |  | 434.x1 | |  |  |  | | 106 | P |  | 86 | 82 (73 to 87) | |  |
|  |  |  |  |  | 433, 434, 436 | |  |  |  | | 175 | P |  | 106 | 61 (53 to 68) | |  |
|  |  |  |  |  | 434, 436 | |  |  |  |  | 127 | P |  | 104 | 82 (74 to 88) | |  |
| Rosamond  1999 | US | | 45-64 | | 433, 434, 436 | | 9-CM | | H | | 560 | - | Medical Record | 252 | 45 (41 to 49) | | 12 |
|  |  |  |  |  | 434, 436 | |  |  |  | | 294 |  |  | 216 | 73 (68 to 78) | |  |
|  |  |  |  |  | 433 | |  |  |  | | 266 |  |  | 36 | 14 (10 to 18) | |  |
|  |  |  |  |  | 434 | |  |  |  | | 186 |  |  | 143 | 77 (70 to 82) | |  |
|  |  |  |  |  | 436 | |  |  |  | | 108 |  |  | 73 | 70 (58 to 76) | |  |
| Rinaldi  2003 | Italy | | - | | 434, 436 |  | 9 | | H | | 180 | P/S | Inpatient register | 128 | 71 (64 to 77) | | 9 |
|  |  |  |  |  | 434, 436 |  |  |  |  | | 157 | P |  | 119 | 76 (69 to 82) | |  |
|  |  |  |  |  | 436 |  |  |  |  | | 177 | P/S |  | 125 | 71 (64 to 77) | |  |
|  |  |  |  |  | 436 |  |  |  |  | | 154 | P |  | 116 | 75 (68 to 81) | |  |
| Leone  2004 | Italy | | - | | 433 |  | 9 | | H | | 134 | P/S | Inpatient register | 8 | 6 (3 to 11) | | 11 |
|  |  |  |  |  | 433 |  |  | |  | | 89 | P |  | 7 | 8 (4 to 15) | |  |
|  |  |  |  |  | 434 |  |  | |  | | 202 | P/S |  | 176 | 87 (82 to 91) | |  |
|  |  |  |  |  | 434 |  |  | |  | | 188 | P |  | 169 | 90 (85 to 93) | |  |
|  |  |  |  |  | 436 |  |  | |  | | 57 | P/S |  | 40 | 70 (57 to 80) | |  |
|  |  |  |  |  | 434, 436 |  |  | |  | | 259 | P/S |  | 216 | 83 (78 to 87) | |  |
|  |  |  |  |  | 434, 436 |  |  | |  | | 236 | P |  | 205 | 87 (82 to 91) | |  |
|  |  |  |  |  | 433, 434, 436 |  |  | |  | | 393 | P/S |  | 224 | 57 (52 to 62) | |  |
|  |  |  |  |  | 433, 434, 436 |  |  | |  | | 325 | P |  | 212 | 65 (60 to 70) | |  |
| Ellekjaer  1999 | Norway | | ≥ 15 | | 436 | | 9 | | H | | 313 | P/S | Stroke register | 206 | 66 (61 to 71) | | 9 |
|  |  |  |  |  | 434, 436 | |  |  |  |  | 402 |  |  | 261 | 65 (60 to 69) | |  |
|  |  |  |  |  | 436 | |  |  |  |  | 89 |  |  | 55 | 62 (51 to 71) | |  |
| Roumie  2008 | US | | 50-84 | | 433.x1, 434.x1, 436 | | 9-CM | | H | | 150 | P | Medical Record | 127 | 85 (78 to 90) | | 9 |
| Johnsen  2002 | Denmark | | 50-64 | | I63 | | 10 | | H | | 113 | P/S | Medical record | 99 | 88 (80 to 92) | | 10 |
|  |  |  |  |  | I63, I64 | |  |  |  |  | 313 |  |  | 238 | 76 (71 to 80) | |  |
|  |  |  |  | | I64 | |  |  |  |  | 200 |  |  | 139 | 70 (63 to 75) | |  |
| Krarup  2007 | Denmark | | - | | I63, I64 | | 10 | | H | | 138 | - | Medical record | 96^g^ | 70 (61 to 77) | | 9 |
|  |  |  |  |  | I64 | |  |  |  | | 105 | - |  | 64^g^ | 61 (51 to 70) | |  |
| Wright  2012 | UK | | - | | I63 | | 10 | | H | | 190 | P/S | GP record^¶^ | 164 | 86 (81 to 90) | | 10 |
|  |  |  |  |  | I63, I64 | |  |  |  |  | 309 |  |  | 242 | 78 (73 to 83) | |  |
|  |  |  |  |  | I64 | |  |  |  |  | 119 |  |  | 78 | 66 (57 to 73) | |  |
| Tirschwell  2002 | US | | ≥ 20 | | 433.X1, 434.X1, 436^§§^ | | 9-CM | | H+D | | i) 50^¶¶^ | P/S | Medical Record | 45 | 90 (79 to 96) | | 9 |
|  |  |  |  |  | 433.X1, 434.X1, 436^§§^ | |  |  |  | | Ii) 50^¶¶^ | P/S |  | 46 | 91 (81 to 98) | |  |
|  |  |  |  |  | 433.X1, 434.X1, 436 | |  |  |  | | iii) 50^¶¶^ | P |  | 44 | 88 (76 to 94) | |  |
| Wahl  2010 | US | | - | | 433.x1, 434.x1, 436, 437.1, 437.9 | | 9-CM | | H | | 200 | - | Medical record | 175 | 87 (82 to 91) | | 9 |
| Haesbert  2013 | France | | >18 | | I63 | | 10 | | H | | 329 | P | Hospital register and medical record | 313 | 95 (92 to 97) | | 11 |
| **Tonolen*  *2007* | *Finland* | | *25-74* | | *433, 434, I63* | | *9 +10* | | *H+D* | | *2711* | *P/S* | *Hospital register* | *2223* | *82 (81 to 83)* | | *5* |
|  |  |  |  |  | *433, 434, 436, I63, I64* | |  | |  | | *2900* |  |  | *2407* | *83 (82 to 84)* | |  |

| **Studies validating codes for haemorrhagic stroke (SAH or ICH)** | | | | | | | | | | | |  |
| --- | --- | --- | --- | --- | --- | --- | --- | --- | --- | --- | --- | --- |
| Rosamund^33^  1999 | US | 45-64 | 430, 431 | | 9-CM | H | 63 | P/S | Medical Record | 46 | 73 (61 to 82) | 12 |
| Leone  2004 | Italy | - | 430, 431 | | 9 | H | 152 | P/S | Inpatient Register | 131 | 86 (80 to 91) | 11 |
| Ellekjaer  1999 | Norway | ≥ 15 | 430, 431 | | 9 | H | 69 | P/S | Stroke Register | 51 | 74 (62 to 83) | 9 |
| Tonolen  2007 | Finland | 25-74 | 430, 431, I60, I61 | | 9+10 | H+D | 729 | P/S | Hospital Register | 646 | 89 (86 to 91) | 5 |
| Johnsen  2002 | Denmark | 50-64 | I60, I61 | | 10 | H | 65 | P/S | Medical Record | 42 | 65 (52 to 75) | 10 |
| **Studies validating codes for subarachnoid Haemorrhage (SAH)** | | | | | | | | | | | |  |
| Tirschwell  2002 | US | ≥ 20 | 430^§§^ | 9-CM | | H+D | i) 51^¶¶^ | P/S | Medical Record | 43 | 86 (72 to 92) | 9 |
|  |  |  | 430^§§^ |  |  |  | ii) 51^¶¶^ | P/S |  | 46 | 89 (79 to 97) |  |
|  |  |  | 430 |  |  |  | iii)51^¶¶^ | P |  | 48 | 94 (84 to 98) |  |
| Tonolen  2007 | Finland | 25-74 | 430, I60 | 9 + 10 | | H+D | 253 | P/S | Stroke Register | 220 | 87 (82 to 91) | 5 |
| Kirkmann  2009 | UK | - | I60 | 10 | | H | 1169 | P | Discharge summary | 1123 | 96 (95 to 97) | 8 |
| Wright  2012 | UK | - | I60 | 10 | | H | 78 | P/S | GP record^¶^ | 75 | 96 (89 to 99) | 10 |

| **Studies validating codes for intracerebral haemorrhage (ICH)** | | | | | | | | | | |  |
| --- | --- | --- | --- | --- | --- | --- | --- | --- | --- | --- | --- |
| Leone^49^  2004 | Italy | - | 431 | 9 | H | 110 | P/S | Inpatient register | 82 | 75 (66 to 82) | 11 |
|  |  |  |  |  |  | 102 | P |  | 78 | 76 (67 to 84) |  |
| Ellekjaer  1999 | Norway | ≥ 15 | 431 | 9 | H | 56 | P/S | Stroke Register | 40 | 71 (59 to 82) | 9 |
| Tonolen  2007 | Finland | 25-74 | 431, I61 | 9 + 10 | H + D | 476 | P/S | Stroke Register | 413 | 87 (83 to 90) | 5 |
| Kirkmann  2009 | UK | - | I61 | 10 | H | 978 | P | Discharge summary | 938 | 96 (94 to 97) | 8 |

PPV: Positive Predictive Value; H: Hospital data; D: Death certificates; H+D: both; P: Primary position code; P/S: Primary or Secondary position code.

**Italics* indicate studies using miscellaneous groups of codes or excluding SAH, not included in Fig. 3.

^†^Number of ICD coded events compared against the reference standard.

^‡^Population register: population based stroke register, Hospital register: inpatient and outpatient stroke register, Inpatient register: inpatient stroke register. Medical Record: definite or probable stroke diagnoses confirmed by review of medical records (excludes ‘possible’ stroke). Medical records were reviewed by stroke physicians or neurologists, unless otherwise specified.

^§^Quality score (total 14). See S1_Appendix for questions and scoring methods.

^¶^Medical record reviewed by ‘cardiovascular researchers’.

^**^Medical record reviewed by ‘trained data abstractors’.

^††^431, 432, 434, 436 primary position, or rehabilitation code primary position AND 430-438 secondary position, OR 433, 434 primary position AND 430-438 secondary position.

^‡‡^Mean value calculated from published data.

^§§^If > 1 code per discharge they were chosen in the following hierarchy: SAH>ICH>IS>TIA

^¶¶^One code for each discharge chosen from: i) First 9 discharge diagnoses ii) First 2 discharge diagnoses iii) Primary discharge code chosen

^***^Abstracts of the medical record reviewed by ‘study physician’.
