# Supplementary material for: Accuracy of Electronic Health Record Data for Identifying Stroke Cases in Large-Scale Epidemiological Studies: A Systematic Review from the UK Biobank Stroke Outcomes Group
Source: PLoS One. 2015 Oct 23;10(10):e0140533. doi: 10.1371/journal.pone.0140533 (PMC4619732; doi:10.1371/journal.pone.0140533)
Supplement: S2 Table — (DOCX) [file pone.0140533.s003.docx]

**S2 Table. Quality assessment of included studies.**

| **Study** | **Reporting** | | | | | | **Generalisability** | | | | **Risk of bias** | | | | | | | **Total score** |
| --- | --- | --- | --- | --- | --- | --- | --- | --- | --- | --- | --- | --- | --- | --- | --- | --- | --- | --- |
|  | Selection Criteria^*^ | Index test^†^ | Reference standard^‡^ | Participants excluded^§^ | Unclear results^¶^ | Total  (5) | Country^**^ | Population selected^††^ | Reference standard^‡‡^ | Total  (3) | Selection bias^§§^ | Blind^¶¶^ | Indep^***^ | Differential verification^†††^ | Reference standard^‡‡‡^ | Timing^§§§^ | Total  (6) | (14) |
| Reker [32] | ☺ | ☺ | ☹ | ☺ | ☹ | 3 | ☹ | ? | ? | 0 | ☹ | ☹ | ☺ | ☺ | ? | ☺ | 3 | 6 |
| Aboa-Eboule [54] | ☺ | ☺ | ☺ | ☺ | ☹ | 4 | ☹ | ? | ☺ | 1 | ☺ | ☺ | ☺ | ☺ | ☺ | ☺ | 6 | 11 |
| Sinha [56] | ☺ | ☺ | ☺ | ☺ | ☺ | 5 | ☺ | ? | ☺ | 2 | ☹ | ☹ | ☺ | ☺ | ☺ | ☺ | 4 | 11 |
| Wahl [41] | ☺ | ☹ | ☹ | ☺ | ☺ | 3 | ☹ | ☺ | ? | 1 | ☺ | ☺ | ☺ | ☺ | ? | ☺ | 5 | 9 |
| Rinaldi [52] | ☺ | ☺ | ☺ | ☺ | ☹ | 4 | ☹ | ☺ | ☺ | 2 | ☹ | ☹ | ☺ | ☹ | ☺ | ☺ | 3 | 9 |
| Benesch [43] | ☹ | ☺ | ☺ | ☹ | ☹ | 2 | ☹ | ? | ? | 0 | ? | ☹ | ☺ | ☺ | ? | ☹ | 2 | 4 |
| Klatsky [39] | ☺ | ☺ | ☺ | ☺ | ☺ | 5 | ☹ | ? | ☺ | 1 | ☹ | ☹ | ☺ | ☺ | ☺ | ☺ | 4 | 10 |
| Goldstein [42] | ☺ | ☺ | ☹ | ☺ | ☹ | 3 | ☹ | ? | ? | 0 | ☹ | ☹ | ☺ | ☺ | ☺ | ☺ | 4 | 7 |
| Koster [64] | ☺ | ☺ | ☺ | ☺ | ☺ | 5 | ☹ | ☺ | ☺ | 2 | ☹ | ☺ | ☺ | ☺ | ☺ | ☺ | 5 | 12 |
| Hasan [62] | ☺ | ☺ | ☺ | ☺ | ☹ | 4 | ☺ | ☹ | ? | 1 | ☺ | ☹ | ☺ | ☺ | ? | ☺ | 4 | 9 |
| Derby [35] | ☺ | ☺ | ☺ | ☺ | ☺ | 5 | ☹ | ☺ | ☺ | 2 | ☹ | ☹ | ☺ | ☺ | ☺ | ☺ | 4 | 11 |
| Derby [36] | ☺ | ☺ | ☺ | ☺ | ☺ | 5 | ☹ | ☺ | ☺ | 2 | ☹ | ☹ | ☺ | ☺ | ☺ | ☺ | 4 | 11 |
| Rosamond [33] | ☺ | ☹ | ☺ | ☺ | ☺ | 4 | ☹ | ☺ | ☺ | 2 | ☺ | ☺ | ☺ | ☺ | ☺ | ☺ | 6 | 12 |
| Barer [60] | ☺ | ☹ | ☹ | ☹ | ☹ | 1 | ☺ | ? | ☺ | 2 | ? | ☺ | ☺ | ☺ | ☺ | ☺ | 5 | 8 |
| Haesebart [53] | ☺ | ☺ | ☺ | ☺ | ☹ | 4 | ☹ | ☺ | ☺ | 2 | ☺ | ☹ | ☺ | ☺ | ☺ | ☺ | 5 | 11 |
| Johnsen [44] | ☺ | ☺ | ☺ | ☺ | ☹ | 4 | ☹ | ☺ | ☺ | 2 | ☹ | ☹ | ☺ | ☺ | ☺ | ☺ | 4 | 10 |
| Appelros [45] | ☺ | ☹ | ☺ | ☺ | ☹ | 3 | ☹ | ☺ | ☺ | 2 | ☺ | ☺ | ☺ | ☺ | ☺ | ☺ | 6 | 11 |
| Tirschwell [40] | ☺ | ☺ | ☹ | ☺ | ☹ | 3 | ☹ | ☺ | ? | 1 | ☺ | ☺ | ☺ | ☺ | ? | ☺ | 5 | 9 |
| Davenport [58] | ☺ | ☺ | ☺ | ☺ | ☺ | 5 | ☺ | ? | ? | 1 | ☺ | ☺ | ☺ | ☹ | ☺ | ☺ | 5 | 11 |
| Stegmayr [50] | ☺ | ☺ | ☺ | ☺ | ☹ | 4 | ☹ | ☺ | ☺ | 2 | ☺ | ☺ | ☺ | ☺ | ☺ | ☺ | 6 | 12 |
| Liu [37] | ☺ | ☺ | ☺ | ☹ | ☹ | 3 | ☹ | ? | ☺ | 1 | ? | ☹ | ☺ | ☺ | ☺ | ☺ | 4 | 8 |
| Kirkman [63] | ☺ | ☺ | ☺ | ☹ | ☹ | 3 | ☺ | ☺ | ☹ | 2 | ? | ☹ | ☺ | ☺ | ☹ | ☺ | 3 | 8 |
| Palmieri [55] | ☺ | ☺ | ☺ | ☹ | ☹ | 3 | ☹ | ☺ | ? | 1 | ? | ☹ | ☺ | ☺ | ☺ | ☺ | 4 | 8 |
| Mant [59] | ☺ | ☺ | ☺ | ☺ | ☹ | 4 | ☺ | ? | ☺ | 2 | ☺ | ☺ | ☺ | ☹ | ☺ | ☺ | 5 | 11 |
| Mayo [38] | ☺ | ☺ | ☹ | ☺ | ☺ | 4 | ☹ | ? | ? | 0 | ☺ | ☹ | ☺ | ☺ | ? | ☺ | 4 | 8 |
| Panayiotou [61] | ☺ | ☺ | ☹ | ☺ | ☹ | 3 | ☺ | ☹ | ☺ | 2 | ☹ | ☹ | ☺ | ☺ | ? | ☺ | 3 | 8 |
| Wright [57] | ☺ | ☺ | ☺ | ☺ | ☺ | 5 | ☺ | ☺ | ? | 2 | ☹ | ☹ | ☺ | ☺ | ? | ☺ | 3 | 10 |
| Sporalore [51] | ☺ | ☺ | ☺ | ☹ | ☹ | 3 | ☹ | ☺ | ☺ | 2 | ? | ☹ | ☺ | ☺ | ☺ | ☺ | 4 | 9 |
| Roumie [34] | ☺ | ☺ | ☺ | ☺ | ☹ | 4 | ☹ | ? | ☺ | 1 | ☹ | ☹ | ☺ | ☺ | ☺ | ☺ | 4 | 9 |
| Krarup [46] | ☺ | ☹ | ☺ | ☺ | ☹ | 3 | ☹ | ☺ | ☺ | 2 | ☹ | ☺ | ☺ | ☺ | ? | ☺ | 4 | 9 |
| Harriss [65] | ☺ | ☺ | ☺ | ☺ | ☹ | 4 | ☹ | ☺ | ☺ | 2 | ☺ | ☺ | ☺ | ☺ | ☺ | ☺ | 6 | 12 |
| Ellekjaer [48] | ☺ | ☺ | ☺ | ☹ | ☹ | 3 | ☹ | ☺ | ☺ | 2 | ? | ☹ | ☺ | ☺ | ☺ | ☺ | 4 | 9 |
| Tonolen [47] | ☺ | ☺ | ☹ | ☹ | ☹ | 2 | ☹ | ☺ | ? | 1 | ? | ☹ | ☺ | ? | ? | ☺ | 2 | 5 |
| Leone [49] | ☺ | ☺ | ☺ | ☺ | ☹ | 4 | ☹ | ☺ | ☺ | 2 | ☺ | ☹ | ☺ | ☺ | ☺ | ☺ | 5 | 11 |
| Ives [29] | ☺ | ☹ | ☺ | ☹ | ☹ | 2 | ☹ | ☺ | ☺ | 2 | ? | ☹ | ☺ | ☺ | ☺ | ☺ | 4 | 8 |
| Lakshminar. [30] | ☺ | ☺ | ☺ | ☹ | ☹ | 3 | ☹ | ☺ | ☺ | 2 | ? | ☹ | ☺ | ☺ | ☺ | ☺ | 4 | 9 |
| Leibson [31} | ☺ | ☺ | ☺ | ☺ | ☹ | 4 | ☹ | ☺ | ☺ | 2 | ☺ | ☺ | ☺ | ☹ | ☺ | ☺ | 5 | 11 |

☺High reporting quality/High generalizability/Low risk of bias ☹ Low reporting quality/Low generalizability/High risk of bias ? Unclear generalizability/Unclear risk of bias.

Rules for assessment of study quality are displayed in S1 Appendix.

*Were the selection criteria clearly described?

†Was execution of the index test described in sufficient detail to allow replication of the test?

‡Was execution of the reference standard described in sufficient detail to permit its replication?

§If participants were excluded from the final analysis, were they described and were the reasons for this exclusion explained?

¶Were uninterpretable/intermediate results reported?

**Was the study UK-based?

††Was the spectrum of patients selected representative of the patients who will receive the diagnosis in practice?

‡‡Were the same clinical data available when test results were interpreted as would be available when used in practice?

§§Did the whole sample, or a random selection of the sample, receive verification using a reference standard of diagnosis?

¶¶Were the reference standard results interpreted without knowledge of the results of the index test?

***Was the reference standard independent of the index test?

†††Did all patients receive the same reference standard regardless of the index test result?

‡‡‡ Is the reference standard likely to correctly classify the target condition?

§§§Was the time period between the reference standard and the index test short enough to be reasonably sure that the target condition did not change between the two tests?
