# Supplementary material for: Accuracy of Electronic Health Record Data for Identifying Stroke Cases in Large-Scale Epidemiological Studies: A Systematic Review from the UK Biobank Stroke Outcomes Group
Source: PLoS One. 2015 Oct 23;10(10):e0140533. doi: 10.1371/journal.pone.0140533 (PMC4619732; doi:10.1371/journal.pone.0140533)
Supplement: S3 Table — (DOCX) [file pone.0140533.s004.docx]

**S3 Table. Sensitivity, Specificity, PPV, and NPV of codes for stroke versus a population reference standard**

| **Study** | **Country** | **ICD code group** | **Code source** | **Reference standard^*^** | | | **Coded events compared against the reference standard^†^ (n)** | | | | **Sensitivity**  **(% & 95% CI)** | **Specificity**  **(% & 95% CI)** | **PPV**  **(% & 95% CI)** | **NPV**  **(% & 95% CI)** |
| --- | --- | --- | --- | --- | --- | --- | --- | --- | --- | --- | --- | --- | --- | --- |
|  |  |  |  | ***Stroke*** | | ***No stroke*** | ***TP*** | ***FP*** | ***TN*** | ***FN*** |  |  |  |  |
| Appelros | Sweden | I61, I63, I64 | D | 377 | | 123,126 | 78 | 20 | 123,106 | 299 | 21 (71-25) | 99.9 (99.97-99.98) | 80 (71-86) | 99.7 (99.75-99.78) |
|  |  |  | H |  | | | 318 | 10 | 123,116 | 59 | 84 (80-88) | 99.9 (99.98-99.99) | 97 (94-98) | 99.9 (99.93-99.96) |
|  |  |  | H + D |  | | | 333 | 30 | 123,096 | 44 | 88 (85-91) | 99.9 (99.96-99.98) | 92 (88-94) | 99.9 (99.95-99.97) |
| Koster | Sweden | I61, I63, I64 | D | 1,351 | 508,649 | | 40 | 62 | 508,587 | 1,311 | 3 (2-4) | 99.9 (99.98-99.99) | 39 (30-49) | 99.7 (99.73-99.76) |
|  |  |  | H |  |  |  | 1224 | 202 | 508,447 | 127 | 91 (89-92) | 99.9 (99.95-99.97) | 86 (84-88) | 99.9 (99.97-99.98) |
|  |  |  | H + D |  |  |  | 1264 | 264 | 508,385 | 87 | 94 (92-95) | 99.9 (99.94-99.95) | 83 (81-85) | 99.9 (99.98-99.99) |
| Stegmayr | Sweden | 430-438 | D  H | 4528 | 94,556 | | 812  3492 | 87  1,609 | 94,469  92,947 | 3,716  1,036 | 18 (17-19)  78 (76-78) | 99.9 (99.88-99.92)  98.3 (98.21-98.38) | 90 (88-92)  69 (67-70) | 96.2 (96.09-96.33)  98.9 (98.83-98.96) |
| Ellekjaer | Norway | 430-438  430,431,434,436 | H  H | 430 | 69,570 | | 369  347 | 390  161 | 69,180  69,409 | 61  83 | 86 (82-89)  81 (77-84) | 99.4 (99.38-99.49)  99.7 (99.73-99.80) | 49 (45-52)  68 (64-72) | 99.9(99.88-99.93)  99.9 (99.85-99.90) |

D: death certificates; H: hospital data; H + D: both

^*^Population stroke register

^†^TP=True Positive cases; FP=False Positive cases; TN=True Negative cases; FN= False Negative cases.
