# Supplementary material for: Accuracy of Electronic Health Record Data for Identifying Stroke Cases in Large-Scale Epidemiological Studies: A Systematic Review from the UK Biobank Stroke Outcomes Group
Source: PLoS One. 2015 Oct 23;10(10):e0140533. doi: 10.1371/journal.pone.0140533 (PMC4619732; doi:10.1371/journal.pone.0140533)
Supplement: S4 Table — (DOCX) [file pone.0140533.s005.docx]

**S4 Table. Influence of diagnostic position on PPV**

| **Study** | **ICD code group** | **Diagnostic position** | **Coded events (n)** | **PPV**  **(% & 95% CI)** |
| --- | --- | --- | --- | --- |
| Leone [49]  2004 | 430-438 | Primary or Secondary | 1017 | 60 (57-63) |
|  |  | Primary | 833 | 66 (63-69) |
| Lakshminarayan [30]  2009 | 431, 432, 434, 436, 437 | Primary or Secondary | 6032 | 63 (61-64) |
|  |  | Primary | 4445 | 85 (84-86) |
| Leibson [31]  1994 | 430-438 | Primary or Secondary | 462 | 54 (49-58) |
|  |  | Primary | 377 | 60 (55-64) |
| Roumie [34]  2008 | 430, 431, 433.x1,434.x1, 436 | Primary or Secondary  Primary | 231  203 | 85 (84-92)  97 (93-98) |
| Liu [37]  1999 | 430-438 | Primary or Secondary | 862 | 56 (53-60) |
|  |  | Primary | 621 | 87 (63-70) |
| Rinaldi [52]  2003 | 434, 436 | Primary or Secondary | 180 | 71 (64-77) |
|  |  | Primary | 157 | 76 (69-82) |
| Tirschwell [42]  2002 | 430 | Primary or Secondary* | 51^*^ | 86 (74-93) |
|  |  | Primary* | 51^*^ | 94 (84-98) |
| Benesch [43]  1997 | 433, 434, 436 | Primary or Secondary | 550 | 43 (39-47) |
|  |  | Primary | 379 | 53 (48-58) |

^*^A single code was selected from 51 hospital discharges: Primary or Secondary: used the hierarchy SAH>ICH>IS>TIA to select a single code for each discharge; Primary: selected the primary discharge code.
