# Supplementary material for: Accuracy of Electronic Health Record Data for Identifying Stroke Cases in Large-Scale Epidemiological Studies: A Systematic Review from the UK Biobank Stroke Outcomes Group
Source: PLoS One. 2015 Oct 23;10(10):e0140533. doi: 10.1371/journal.pone.0140533 (PMC4619732; doi:10.1371/journal.pone.0140533)
Supplement: S5 Table — (DOCX) [file pone.0140533.s006.docx]

**S5 Table. Included primary care Read-coded studies: characteristics and results**

| **Study** | **Country** | **Age**  **(range)** | **Read code(s) assessed** | **Coded events assessed (n)** | **Diagnosis sought** | **Coded events confirmed (n)** | **Reference standard** | **PPV**  **(% & 95% CI)** |
| --- | --- | --- | --- | --- | --- | --- | --- | --- |
| Rugiomez ^[27]^  2010* | UK | 40-84 | G66..00 to G669.00^†^ | 120 | Ischaemic stroke | 93 | GP questionnaire/ GP record | 78 (69 to 85) |
|  |  |  | G64..00 to G64z.00^‡^ | 28 | Ischaemic stroke | 25 |  | 89 (72 to 98) |
| Gaist ^[28]^  2013^§^ | UK | 20-89 | 41 codes^¶^ | 306 | ICH & SAH | 251 | GP questionnaire/ GP record | 82 (77 to 86) |
|  |  |  |  | 156 | SAH | 142 |  | 91 (85 to 95) |
|  |  |  |  | 150 | ICH | 109 |  | 73 (65 to 80) |

**^*^**This study excluded individuals who were not admitted to hospital, who had cancer or a previous cerebrovascular event, or who also had Read codes for haemorrhagic stroke.

^†^Read codes for unspecified stroke.

^‡^Read codes for ischaemic stroke.

^§^This study excluded Read code events if the primary care record ‘free text’ suggested that events were; ischaemic; secondary to trauma; subdural haemorrhage; prevalent rather than incident; due to cancer, or events occurred in hospital.

^¶^Diagnostic codes for Subarachnoid haemorrhage (SAH), Intracerebral haemorrhage (ICH), unspecified haemorrhage and sequelae, Procedure codes for evacuation/aspiration of haematoma, and Process of care codes for ‘History of subarachnoid haemorrhage.’ Gaist et.al, Pharmacoepidemiology and Drug Safety 2013;22:176-182:Appendix I (online supplement).
